# Effectiveness of a motivated, action-based intervention on improving physical activity level, exercise self-efficacy and cardiovascular risk factors of patients with coronary heart disease in Sri Lanka: A randomized controlled trial protocol

**DOI:** 10.1371/journal.pone.0270800

**Published:** 2022-07-05

**Authors:** Karthikesu Karthijekan, Ho Yu Cheng

**Affiliations:** 1 The Nethersole School of Nursing, Faculty of Medicine, The Chinese University of Hong Kong, Hong Kong SAR, China; 2 Department of Supplementary Health Sciences, Faculty of Health-Care Sciences, Eastern University, Batticaloa, Sri Lanka; Kurume University School of Medicine, JAPAN

## Abstract

**Background:**

Coronary heart disease (CHD), is the major contributor to cardiac-associated mortality worldwide. Lifestyle modification, including physical activity, is highly recommended for secondary prevention for patients with CHD. However, many people in Sri Lanka with CHD do not engage in adequate physical activity.

**Objective:**

To develop a culture-specific, motivated, and action-based intervention and examine its effects on physical activity level, exercise self-efficacy, and cardiovascular risk factors among patients with CHD.

**Methods and materials:**

This is an assessor-blinded randomized controlled trial that will recruit 150 patients with CHD from the inpatients cardiac unit of a hospital in Batticaloa, Sri Lanka, and will randomly assign them either to the intervention group or the control group. The participants in the intervention group will receive a culture-specific and motivated, action-based intervention in addition to the usual care, while participants in the control will only receive the usual care. The intervention consists of a face-to-face preparatory session and 12-week motivated, action-based sessions which were developed based on the health action process approach (HAPA) framework. The face-to-face preparatory session will identify the health needs of the participants, develop a goal-oriented patient-centered action plan, and provide knowledge and an overview of the program. The 12-week motivated, action-based sessions consist of three monthly group education and center-based group exercises, followed by three 20-min individualized telephone follow-ups. Outcomes will be assessed immediately after the intervention and at one-month post-intervention.

**Discussion:**

This protocol proposes a supervised centered-based group exercise with group education, and individualized telephone follow-ups guided by the HAPA framework to improve the physical activity level, exercise self-efficacy, and cardiovascular risk factors of patients with CHD. Results from this study will inform the effectiveness of a motivated, action-based intervention in a low-resource setting and provide information on the feasibility, barriers, and facilitators for lifestyle modification in Sri Lanka.

**Trial registration:**

ClinicalTrial.gov.org PRS: NCT05051774; Date of registration: September 21, 2021.

## Introduction

Coronary heart disease (CHD), a major group of cardiovascular disorders (CVDs), is one of the major contributors to cardiac-associated mortality worldwide, including in Sri Lanka which, causes 8.9 million deaths worldwide in 2019 [1, 2], and an evolving health issue in developing countries; its mortality is declining around the globe [3]. In low-middle income countries like Sri Lanka, death and premature death associated with CHD in all age groups increased by 15.6% and 5% from 2009 to 2019 [4]. Modifiable risk factors, such as physical inactivity, smoking, consumption of an unhealthy diet and alcohol, elevated body mass index (BMI), high blood pressure, elevated serum lipids, and elevated fasting blood glucose levels are common among patients with CHD [5, 6]. Prolonged exposure to these preventable risk factors increases the risk of recurrent cardiac events and premature death and leads to poor prognosis among patients with CHD [7]. CHD is one of the major contributors to disability-adjusted life years (DALYs), and the total number of CHD-associated DALYs has gradually increased since 1990, reaching 182 million in 2019 [8].

Cardiac rehabilitation (CR), a well-established recommendation for secondary prevention in patients with CHD, comprises six core components, namely, health behavior change and education, lifestyle risk factor management, psychosocial health, medical risk management, long-term strategies, and audit and evaluation. CR is effective in reducing all-cause mortality, cardiac mortality, myocardial re-infarction, and cardiovascular risk factors and improving physical activity and quality of life in high, low, and middle-income countries [9, 10]. Although comprehensive CR is effective in improving the health outcomes of patients with CHD, its availability worldwide is only 38% because of inadequate physical and financial resources [11]. Providing a center-based CR is particularly difficult in South Asia including Sri Lanka because of the lack of human resources, space and equipment, and financial support [11, 12]. Therefore, effective strategies for the secondary prevention of CHD are urgently needed.

Lifestyle modification, including physical activity, is one of the key recommendations for secondary prevention for patients with CVDs [13]. A systematic review and meta-analysis of 85 randomized controlled trials (RCTs) that included people with CHD reported that engaging in physical activity contributes to a substantial reduction in all-cause mortality, cardiovascular mortality, recurrent cardiac events, and improvements in health-related quality of life (HRQoL) [14]. However, inadequate physical activity is common among people with CHD in both developed and developing countries, and its prevalence ranges from 56.7% (Sri Lanka) to 83% (United States) [15, 16].

With the increasing morbidity and mortality rate from CHD, especially in low- and middle-income countries, secondary prevention including exercise-based CR plays an important role in improving the prognosis of patients with CHD [7, 17]. High prevalence of physical inactivity, unhealthy dietary practices, poor control of blood glucose, blood pressure (BP), blood lipid, and body weight (BW) was found among patients with CHD in the world, as well as in Sri Lanka [16, 18–20]. Therefore, a culturally appropriate intervention must be designed and implemented using a theoretical framework to improve the physical activity level, exercise self-efficacy, and cardiovascular risk factors in patients with CHD in Sri Lanka.

### Health action process approach (HAPA)

The intervention is underpinned by the HAPA framework to enhance an individual transitioning the intention into action. HAPA is a social-cognitive framework specifying motivational and volitional determinants of health behavior [21], and it is used to guide various health promotion interventions, such as promoting adequate physical activity [22] and dietary behaviors [23]. HAPA comprises two phases, namely, the motivation phase and the volition phase, which emphasizes the initiation and maintenance of health behaviors.

HAPA uses a patient-centered collaborative approach that assesses patients’ beliefs on their health problems experienced, their confidence to perform the assigned action, and their expectations to initiate the intention and form target goals and action plans for behavioral change. Inter-active teaching and exercise can help the participants enhance their knowledge and skills to improve their confidence to transform the intention into action. Individual confidence is also important in dealing with barriers that arise during behavioral action, such as resuming difficult behavior after an interruption and helping continue the achieved desired behavior [21].

## Methods and materials

### Aims and study design

This assessor-blinded, two-arm parallel RCT aims to develop a culture-specific, motivated, and action-based intervention and examine its effects on physical activity level, exercise self-efficacy, and cardiovascular risk factors of patients with first-onset CHD in Sri Lanka.

### Study setting

The study will be conducted at the cardiology clinic of the university-affiliated Teaching Hospital Batticaloa, Sri Lanka, where patients with CHD are treated and discharged under stable medications or referred to another hospital for further conservative management such as percutaneous coronary intervention or coronary artery bypass graft. There are around 360 newly diagnosed patients with CHD are admitted to this hospital each year.

Study participants will be patients newly diagnosed with CHD that are admitted to the cardiac unit and medical wards of the Teaching Hospital Batticaloa, Sri Lanka. The intervention will be delivered at the study hospital’s cardiology clinic. All CHD patients of the study clinic will receive multidisciplinary services including monthly medical check-ups, unstructured health education on a healthy lifestyle, and a brief session on managing diseases, risk factors, and stress provided by a team including cardiologists, physicians, nurses, and other health care workers. Cardiac patients attending other OPD services are also referred to the cardiac clinic for health assessment and unstructured health education.

### Study participants/eligibility criteria

Eligibilities of the participants are adults aged 18 years and above, hospitalized with CHD for the first time, diagnosis confirmed by electrocardiogram or angiography within 6 months, able to read and speak Tamil, obtained a medical clearance from a cardiologist to perform the exercise, and no prescribed activities or exercise restriction. The exclusion criteria are as follows: absolute and relative contradictions to exercise testing and training or at high risk for exercise prescription according to the American College of Sports Medicine guideline and American Association of Cardiovascular and Pulmonary Rehabilitation guideline [24]; any diagnosis of life-limiting conditions; a diagnosis of acute psychotic disease; and unable to independently perform physical activities. Eligible participants will be identified by the research nurse from the coronary care unit and medical wards of the study hospital by reviewing their medical records. In addition, the cardiologist and physiotherapist at the study hospital will be consulted to confirm the suitability of the participants that will perform the interventional exercise.

### Sample size estimation

The sample size estimation is based on the primary outcomes of the study, namely, the physical activity level. Referring to the findings of the literature review, the effect size on improving physical activity is from 0.5 [25] to 0.8 [26]. Therefore, the smallest effect size of 0.5 [25] will be considered in calculating the sample size for this study. To achieve a power of 1-β = 0.8 with a significance level of 0.05 (two sides) and account for 14% of the attrition rate [27] of the previous study, a total of 150 participants will be recruited in this study.

### Randomization, blinding, and data collection procedure

**Fig 1:**
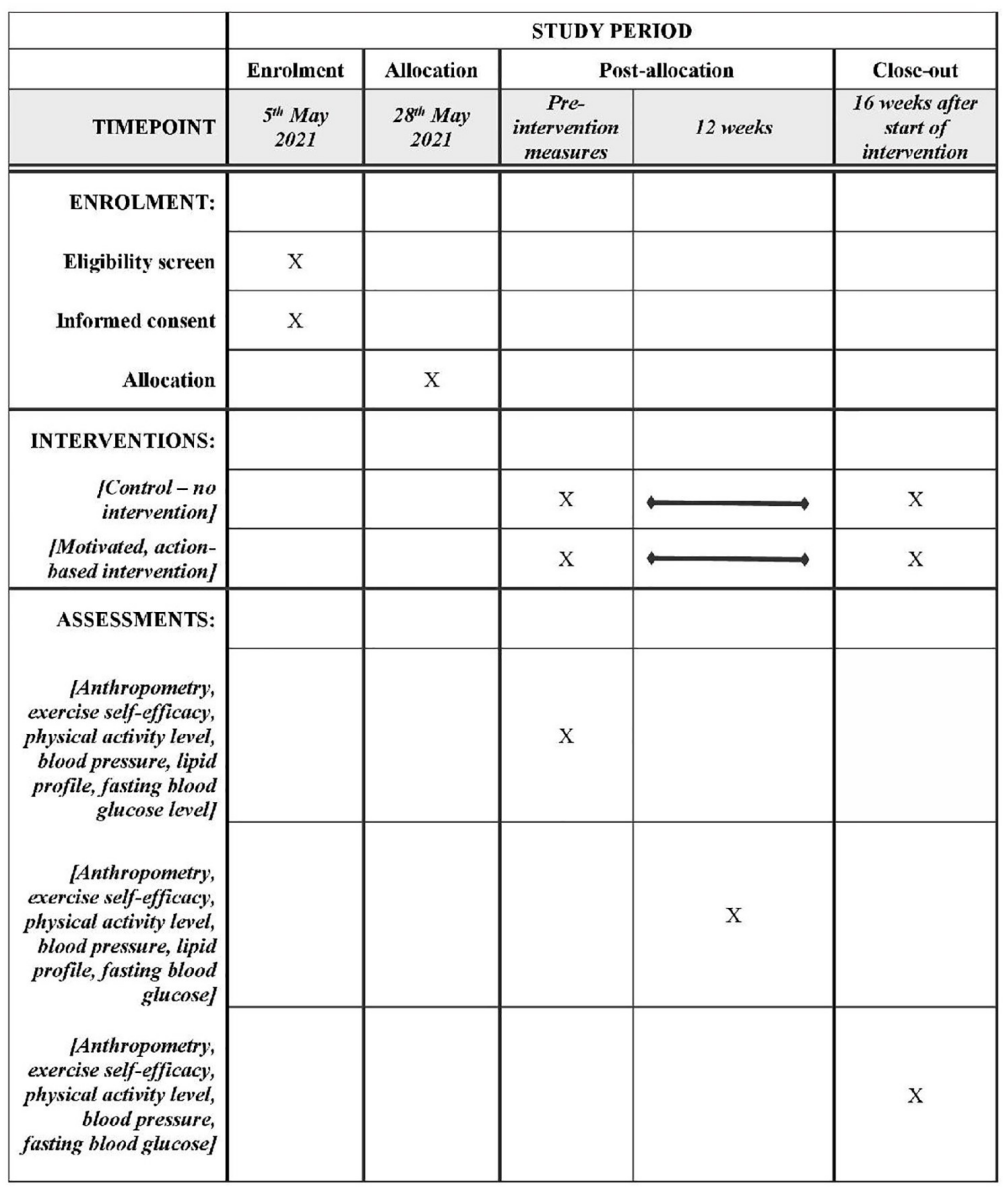
SPIRIT schedule of enrolment, intervention, and assessment of the outcomes.

**Fig 2:**
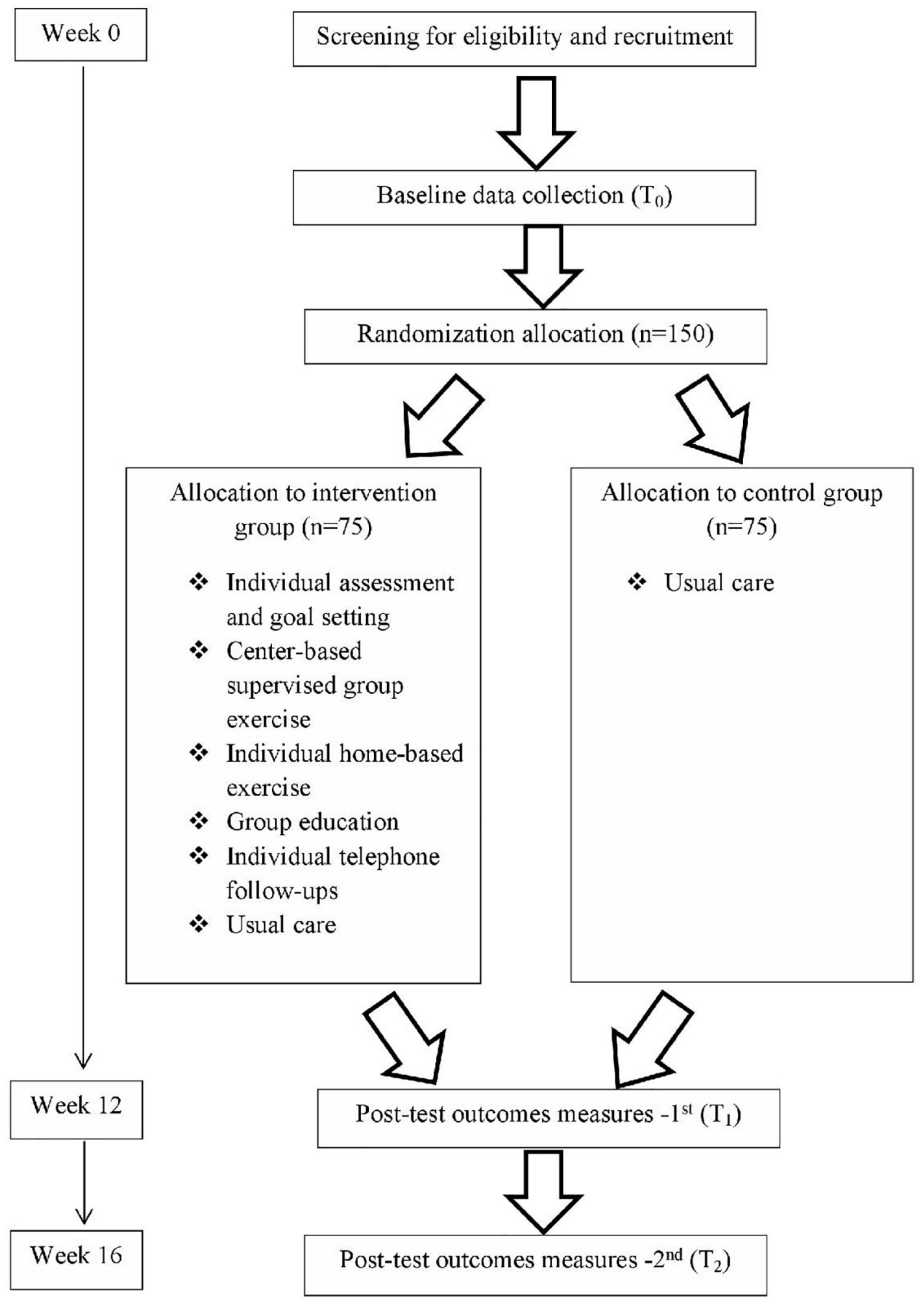
Flow diagram of the study protocol.

All eligible participants will be invited to participate in this study, and a thorough explanation of the study by using the information sheet will be provided by the researcher (Fig 1). Informed consent will be obtained from the participants by the researcher before the study begins. All eligible participants will be classified as either male or female because gender influences the level of physical activities among patients with CHD [28]. The participants will be randomized either to an intervention or control group with a ratio of 1:1 using block randomization with a block size of eight. Random sequencing for group allocation will be generated by an online application (http://www.randmization.com). The group allocation will be placed into an opaque sealed envelope and will be given to the participants after baseline data collection (T_0_) by the research assistant who is independent of the study. The participants will not be blinded to their group allocation because of the nature of the study. The outcomes will be evaluated after completiing the 12-week intervention (T_1_) and one-month post-intervention (T_2_) by a trained research assistant blinded to the participants’ allocation (Fig 2). The outcomes that will be evaluated are the physical activity level, exercise self-efficacy, and cardiovascular risk factors. Every participant will receive 230 Sri Lankan rupees (1 United States Dollar) after completing the data collection to compensate for their time spent on data collection.

### Motivated action-based intervention

To initiate and maintain the positive behavioral changes and health outcomes of patients with CHD, who are discharged from the study hospital, the motivated action-based intervention is developed according to the HAPA framework. The intervention will be provided in two phases: 1) a brief face-to-face preparatory phase, and 2) a 12-week intervention phase using the HAPA framework. The researcher, a registered nurse with extensive experience in clinical teaching and patient education, will be involved in delivering the intervention with support from the cardiologist, medical doctor, cardiac nurses, and physiotherapist to optimize the patients’ outcomes.

#### Face-to-face preparatory phase

The preparatory phase includes one individualized health counseling session for 60 min and it will be conducted by the researcher at the study hospital’s cardiology clinic within 6 months after the CHD event. During this session, the participant’s health needs will be recognized by assessing the health condition, reviewing the daily activities, exploring the discrepancy between activity levels, and the recommended exercise guidelines, identifying the three desired outcomes (achievable and observable) and forming individualized exercise goals and a behavior-specific action plan for behavioral changes. In addition, knowledge and an overview of the program and real success stories will be shared with the participants.

The self-reported risk of CHD will be compared with the calculated Framingham risk score in identifying the discrepancy in the risk level and enhancing the risk perception of participants. In addition, the participants’ concerns and questions will be addressed in this phase to alleviate their fear and anxiety to perform the exercise and enhance their knowledge of CHD.

#### Intervention phase

The 12-week intervention consisting three components: a) three monthly group education sessions (10 participants in each group), b) three monthly center-based supervised group exercises (10 participants in each group), and c) individualized telephone follow-up will be conducted 1 week after the preparatory phase to transform the intention into action and to maintain the behavioral change. The intervention will be delivered as follows.

*Weeks 1–4*. ***Week one (day one)***: To facilitate an individual’s behavioral change, a 40-min face-to-face group education on nature, risk factors, signs and symptoms, and management of CHD and medication adherence will be delivered by the researcher at the hospital’s cardiology clinic. The educational booklet consisting the following topics: nature of CHD, risk factors, signs and symptoms of CHD, heart-healthy diet, physical activity, quitting smoking, stress management, and medication management and adherence. This booklet was developed based on the updated international guideline and culturally appropriate national recommendation for CHD [29–36] and validated by an expert panel consisting of registered physiotherapists, registered nutritionists, cardiologists, and the cardiac nurse for their safety and appropriateness for patients with CHD in Sri Lanka. This booklet will be given to the participants on day one to provide culturally relevant written resources to encourage the patients to follow the health advice at home. The knowledge gained in the education will help the participants form the intention by increasing risk perception, positive outcome expectancies, and self-efficacy in the motivational phase of HAPA. In addition, the education will support the participants in planning the action of intended behavior and help in maintaining self-efficacy throughout the volitional phase of HAPA.

Apart from the group education, a hospital plus home-based heart-healthy exercise will be implemented by the researcher with the support of a physiotherapist, registered nurse, and medical officer at the study hospital’s cardiology clinic. On day one in the first week of the intervention, participants will be guided to perform the exercise (warm-up exercise, brisk walking, and cool-down exercise) with cultural background music (Raravenu Gopabala Instrumental flute version) to perform the exercise at the prescribed intensity and duration and reduce the rating of perceived exertion during exercise [37, 38] under the supervision of a physiotherapist. As suggested by the cardiologist, the exercise will start at low intensity with cultural background music with a slow tempo (40–60 beats per minute). The participants will be guided to perform the warm-up exercise for 5 min before walking. Then participants will be asked to walk for 20 min with the speed paced by the music tempo of Raravenu Gopabala Instrumental flute version. After completing the walking exercise, the participants will perform the cool-down exercise for 5 min.

The participants will be asked to continue the exercise at home with the same duration and intensity performed on day one in the first week of the intervention at the hospital with culturally background music at least three times per week. On day one, an exercise logbook will be distributed to the participants, and the researcher will teach them how to fill the logbook and how to follow the exercise plan for home-based practice. This exercise log record will allow the participants to self-monitor and track their exercise practice at home to maintain the action and coping plan of the HAPA.

***Week three (days 15–22)***: The researcher or trained research assistant will provide a 20-min telephone follow-up at week three to strengthen the volition of the participant in performing the exercise, following the healthy diet at home, enhancing problem-solving skills in overcoming perceived barriers and providing reinforcement for any resulting positive changes. In each telephone follow-up, the goal and action plan will be reviewed and mutually adjusted as necessary for the next follow-up review.

*Weeks 5*–*8*. ***Week five (day one)***: Forty-minute face-to-face group education on physical activity and a heart-healthy diet will be delivered by the researcher at the hospital’s cardiology clinic on day one of the fifth week of the intervention. Apart from group education, participants will be guided to perform the exercise with the same duration and intensity performed in the first session on day one in the first week of the intervention (warm-up exercise: -5 min, brisk walking: -20 min, and cool-down exercise: -5 min) with cultural background music with a slow tempo under the supervision of a physiotherapist. In addition, participants will be advised to continue the exercise at home with the same duration and intensity performed on day one in the fifth week of the intervention at the hospital with cultural background music at least three times per week. Participants will be asked to complete the exercise logbook to determine the progress of the exercise.

***Week seven (days 43***–***50)***: A 20-min telephone follow-up at week seven will be provided by the researcher or trained research assistant with the same objectives as the telephone follow-up at week three.

*Weeks 9*–*12*. ***Week nine (day one)***: Forty-minute face-to-face group education on how to quit smoking and stress management will be delivered by the researcher at the hospital’s cardiology clinic on day one of the ninth week of the intervention. Apart from the group education, participants will be guided to perform the exercise with moderate intensity (warm-up exercise: -5 min, brisk walking: -30 min, and cool-down exercise: -5 min) with cultural background music under the supervision of a physiotherapist. In addition, participants will be advised to continue the exercise at home with the same duration and intensity performed on day one in the ninth week of the intervention at the hospital with cultural background music at least three times per week until the end of the 12-week intervention. Participants will be asked to complete the exercise logbook to determine the progress of the exercise.

***Week 11 (days 61***–***67)***: A 20-min telephone follow-up at week 11 will be provided by the researcher or trained research assistant with the same objectives as the telephone follow-up at week three.

### Usual care

Participants in both groups will receive the usual care services for patients with CHD provided in the study hospital. For example, an unstructured health education conducted by a nurse on a healthy lifestyle (exercise, heart-healthy diet, stress management, and smoking cessation) will be given once a month for 1 year after discharge through face-to-face sessions. During the first visit of the patient with CHD to the cardiology clinic 2 weeks after discharge, the cardiologist will assess the patient’s condition and provide brief unstructured health education on their conditions, focusing on risk factor management and stress management for 10–15 min. The patients will also be instructed to visit the cardiology clinic every month for medical follow-up.

### Data collection

The primary outcome of this study is the physical activity of the participants, whereas the secondary outcomes are cardiac exercise self-efficacy and cardiovascular risk factors, namely, body weight, BMI, waist-hip ratio (WHR), blood pressure, serum lipid levels, and fasting plasma glucose level. Physical activity, cardiac exercise self-efficacy, body weight, BMI, WHR, blood pressure, and fasting plasma glucose will be measured at baseline, immediate post-intervention, and one-month post-intervention, whereas the lipid profile will be measured at baseline and immediately after the intervention.

#### Socio-demographic and clinical data

Socio-demographic and clinical data will be collected at baseline to identify the factors that may influence the outcomes of this study. The socio-demographic characteristics, including gender, age, marital status, ethnicity, education level, employment status, and monthly family income, will be collected from the participants. Clinical characteristics, including duration of illness, medication history, and other comorbidities, will be collected from their medical records.

#### Primary outcomes

*Physical activity*. The primary outcome of the study is a behavioral change, which is physical activity. Physical activity refers to body movements that result in energy expenditure [39]. The International Physical Activity Questionnaire Short Form (IPAQ-SF) will be used in collecting the self-reported time spent in walking, moderate, and vigorous activities lasting at least 10 min, and time spent in sitting using the seven items [40]. The original English version of IPAQ-SF demonstrated high reliability with test-retest reliability ranging from 0.32 to 0.88 with more than three-quarters of studies showing a value greater than 0.65 [40]. The validated Tamil version of IPAQ-SF will be used in assessing the self-reported time spent in walking, moderate and vigorous-intensity activities, and sitting in this study.

#### Secondary outcomes

*Exercise self-efficacy*. Self-efficacy refers to an individual’s confidence to perform the given task and is a key indicator of behavioral change [41]. Self-efficacy is an important determinant of physical activity, and individuals with higher self-efficacy are likely to engage more in physical activities [42]. Cardiac Exercise Self-Efficacy Instrument (CESEI) will be used to assess the exercise self-efficacy of participants in this study. The original English version of CESEI is a 16-item unidimensional instrument that was developed by Hickey et al., in 1992 to assess an individual’s confidence to perform the exercise in different circumstances in cardiac patients [43]. Each item is rated on a five-point Likert scale from 1 (very little confidence) to 5 (quite a lot of confidence). Participants will be asked to rate their confidence level in performing exercises in various situations such as “fitting exercise into a busy day,” “exercising without getting chest pain,” “taking own heart rate before and after the exercise,” and “enduring light exercise.” The CESEI score ranges from 16 to 80, and a high score indicates a high level of self-efficacy in performing the exercises. The original English version of CESEI demonstrated good internal consistency with Cronbach’s alpha 0.97 and test-retest reliability with an intraclass correlation coefficient of 0.87 in a sample of CR patients [43]. Furthermore, significant known-group validity is found in exercise self-efficacy between marathon runners and CR patients (p = 0.04) [43]. The exercise self-efficacy level of participants will be measured using a validated Tamil version of CESEI.

*Cardiovascular risk factors*. ***Anthropometry***: All anthropometric measurements will be performed by a trained research assistant who will be blinded to the group allocation of participants. The weight will be measured with a calibrated mechanical personal scale (model-CAMRY, model number-BS 20140). Participants will be weighed wearing light clothes, without shoes, and an empty bladder. The height will be measured in a standing position by using a flexible plastic tape (without shoes), and the BMI will be calculated as weight in kilograms divided by height squared in meters (kg/m^2^). Waist circumference will be measured using non-stretchable measuring tape at the approximate midpoint between the lower margin of the last palpable rib and the top of the iliac crest to the nearest 1 cm at the end of normal expiration. Hip circumference will be measured at the widest level over the greater trochanters using a flexible plastic tape to the nearest 0.1 cm. WHR will be estimated as waist measurement (cm) divided by hip measurement (cm).

***Blood pressure***: Blood pressure in a sitting position will be measured using a mercury sphygmomanometer (Dekamet model Code: 0125). Participants will be asked to rest for 15 min and no caffeinated food should be consumed or drink for 30 min before measuring the blood pressure.

***Serum lipid levels***: The participants’ total cholesterol, high-density lipoprotein, low-density lipoprotein, and triglyceride levels will be obtained from their medical records.

***Fasting plasma glucose***: The participants’ fasting plasma glucose levels will be obtained from their medical records.

#### Data analysis

Data will be analyzed using SPSS version 22.0 statistical software with a 5% significance level (two-sided; SPSSINC., CHICAGO, IL, USA). Data will be analyzed using descriptive and inferential statistics. Descriptive statistics with mean, standard deviation, range, frequency, and percentage will be used to describe the participants’ socio-demographic characteristics and clinical data. The normality of the data will be tested using the skewness and kurtosis statistics and graphically analyzed by normal Q-Q plots. An independent t-test will be used to compare the baseline characteristics of the intervention and control group for continuous variables while the Chi-square test will be used for categorical variables. The generalized estimating equation model will be used to test the hypotheses, and determine the significant differences across different time points (effect of time, group, and time x group interaction) with adjustment for potential confounding variables to obtain a more precise estimation of the intervention effect. By clinical judgment and statistical incomparability at baseline, the potential confounders will be selected in this study. Intention-to-treat analysis will be used as an approach in analyzing all participants in the final analysis according to the original assigned group, irrespective of the treatment received. If these assumptions are not met, the equivalent non-parametric statistics will be used to analyze the data.

#### Ethical consideration

Ethical approval was obtained from The Joint Chinese University of Hong Kong–New Territories East Cluster Clinical Research Ethics Committee (CREC Ref.No.: 2020.678-T) and Ethics Review Committee, Faculty of Health Care-sciences, Eastern University, Sri Lanka (E/2021/01). This trial protocol had been registered in ClinicalTrials.gov Protocol Registration and Results System (PRS) (NCT05051774). The study protocol adhered to the principles of the Declaration of Helsinki [44]. The approval was also obtained from the study hospital. The purpose of the study, data collection procedures, potential risks and benefits, maintenance of confidentiality, and voluntary basis of participation will be clearly explained to the participants. Written informed consent of the participants will be obtained from them. To ensure the participants’ confidentiality during the study, their names will be coded, and the codes will be stored separately to ensure anonymity. All electronic data will be stored by the principal investigator in a password-protected computer in a secured place. The paper records will be kept by the principal investigator in a locked cabinet. The personal data will only be accessed by the principal investigator. In addition, entered data will be cross-checked by a second person. All the data collected will be destroyed 5 years after the study is completed.

#### Potential risk management

Participants will be informed that there will be no cost for participating in this study, and it will not replace the health services provided by the healthcare professionals of the study hospital. The participants who refuse to continue the study for any given reason will be allowed to pull back from the study without any cost, and withdrawing from the study would not affect the amount of care they receive from the hospital. Participants will be asked to start the exercise at a low intensity and gradually increase in frequency, duration, and intensity. In addition, for continuing exercise at the patients’ homes, participants will be advised to continue the exercise performed in the previous week at the hospital. Moreover, participants will be encouraged to communicate with the researcher (registered nurse) whenever they need any information on CHD and to report any adverse events. Participants will be provided with an educational booklet about signs and symptoms that may develop during the exercise such as angina, dyspnea, tiredness, nausea, and vomiting, and symptom management information. If they develop any discomfort and have to seek medical help, they will be advised to stop the exercise immediately. Furthermore, an exercise self-evaluation form will be given to them to record their daily exercise in terms of frequency, duration, time, and intensity, and they will also be asked to write their self-perceived ideas about exercise to determine its progress. The emergency card with the research nurse’s name and contact information will be given to all participants for communication if they develop any discomfort due to exercise or disease deterioration.

## Discussion

This study aims to examine the effects of a motivated, action-based intervention consisting of three monthly supervised group exercises, group education, and individualized telephone follow-ups that are developed based on the HAPA framework and integrated with behavioral change strategies including improving exercise, following a healthy diet, and enhancing exercise self-efficacy to 1) improve physical activity, 2) enhance exercise self-efficacy, and 3) improve cardiovascular risk factors in patients with CHD in Sri Lanka. The intervention is developed based on the international recommendation for this population, and its cultural relevance to the Sri Lankan context is ensured using the national cultural appropriate recommendation to this population. This study encourages the participants to be actively involved with healthcare professionals in planning the action plan for behavioral change using the individualized risk assessment, goal setting, and awareness of the discrepancy between recommended guidelines and their daily activities. During their stay at their home, the individualized consultation of participants with the researcher or trained research assistant through telephone calls will serve as a communication platform in clarifying their doubts and supporting them to achieve the targeted goals in behavioral change. This study encourages the participants to share their experiences in behavioral change and support one another to motivate and achieve their goals.

As purported by the HAPA framework that underpinned the intervention of the current study, improving an individual’s exercise self-efficacy could lead to engagement in more physical activities. Self-efficacy plays a key role in behavior modifications, such as improving physical activity [23, 45, 46]. The intervention could enhance the self-efficacy of the intervention group during and after the intervention and might help them to overcome the barriers faced for continuing the physical activity at home. A systematic review of patients with CHD showed that listening to music significantly improved their adherence to exercise, facilitated to maintain the exercise after the intervention, improved exercise capacity, mood, cognition, and reduced perceived exertion [47]. In the current study, cultural music is used to facilitate the exercise of participants, which could help them to engage in more physical activities and maintain it after completing the intervention. In addition, music-faced physical activity improves the maintenance self-efficacy of the HAPA framework, thus could lead to a great improvement in the participants’ physical activities in the intervention group.

Supervised exercise training showed a significant improvement in several cardiometabolic outcomes, such as exercise capacity, body fat, BMI, and level of physical activity compared to non-supervised exercise training [48, 49]. The current study has provided supervised exercise training, which could encourage the participants to adhere to exercise, resulting in great improvement in their physical activity levels. Effective communication via mobile health is an effective approach in facilitating the patients with CHD to adhere to the exercise intervention as the secondary prevention of CHD [50, 51]. In the current study, participants will be communicated through telephone calls to strengthen their volition in performing the exercise at home and enhancing their problem-solving skills to overcome perceived barriers, continue the exercise, and reinforced any resulting positive changes.

### Limitations of the study

This study has some limitations. Although the participants for this study will be recruited from a university-affiliated teaching hospital in a district in Sri Lanka that provides extensive health services to the population with diverse socio-demographic and economic backgrounds, the general application of the findings may be limited because of the limitation of the population size in a certain district in Sri Lanka. Even though the study outcomes include objective blood parameters, there could be a risk for recall bias on the self-reported outcomes, such as exercise self-efficacy, which would be measured by a validated instrument.

## Conclusion

This study will be the first RCT to evaluate the culture-specific, motivated, action-based intervention coordinated by nursing health professionals in improving the physical activity level, exercise self-efficacy, and cardiovascular risk factors among patients with CHD in Sri Lanka. This study provides a unique and culturally relevant information to Sri Lankans and South Asians with similar cultural backgrounds on how to improve the modifiable risk factors among the CHD population. This study provides insights into which level this could be applicable with limited resources in developing counties like Sri Lanka where there is scarcity in accessibility and affordability of the comprehensive CR. In addition, this study informs healthcare professionals about exercise prescriptions for individuals who lack the time to engage in physical activity.

## Supporting information

S1 Checklist SPIRIT checklist. (DOC)

S1 File Study protocol approved by the ethics committee in English. (PDF)

## Abbreviations

AACVPR: American Association of Cardiovascular and Pulmonary Rehabilitation
ACSM: American College of Sports Medicine
BMI: Body mass index
CESEI: Cardiac exercise self-efficacy instrument
CHD: Coronary heart disease
CR: cardiac rehabilitation
CVDs: Cardiovascular disorders
HAPA: Health action process approach
IPAQ-SF: International physical activity questionnaire short form
WHR: Waist hip ratio
