# Supplementary material for: Effectiveness of a motivated, action-based intervention on improving physical activity level, exercise self-efficacy and cardiovascular risk factors of patients with coronary heart disease in Sri Lanka: A randomized controlled trial protocol
Source: PLoS One. 2022 Jul 5;17(7):e0270800. doi: 10.1371/journal.pone.0270800 (PMC9255726; doi:10.1371/journal.pone.0270800)
Supplement: S1 File — (PDF) [file pone.0270800.s002.pdf]

Version – 2

The effectiveness of a motivated, action-based intervention on improving physical activity level, exercise self-efficacy and cardiovascular risk factors of coronary heart disease patients: A randomized controlled trial

Introduction

Cardiovascular disorders (CVDs) are the leading cause of morbidity and mortality and accounts for 31% of deaths globally (World Health Organization [WHO], 2017). Low and middle-income countries are more vulnerable to this global CVDs death. Coronary heart disease (CHD), the major group of CVDs, is the leading cause of cardiac-associated mortality, causing >9 million death in 2016 (American Heart Association [AHA], 2015; WHO, 2018). The CHD mortality remains high in developing countries, although the global mortality is declining (Nowbar, Gitto, Howard, Francis, & Al-Lamee, 2019).

The global prevalence of obesity, hypertension (HT), diabetes mellitus (DM), and hyperlipidemia among the CHD population were 40.2%, 47.2%, 39.5%, and 32.7% respectively (Alhabib et al., 2020), whereas this prevalence was comparable in Sri Lanka except that higher prevalence of hyperlipidemia (37%) among the CHD population of Sri Lanka (Jayawardena et al., 2016; Ralapanawa et al., 2019). These modifiable risk factors increase the risk of recurrent cardiac events, premature death, and lead to poor prognosis among CHD patients (Zengin et al., 2015).

Cardiac rehabilitation (CR), comprises the six core components namely, health behavior change and education, lifestyle risk factor management, psychosocial health, medical risk management, long-term strategies, and audit and evaluation had shown to be effective in reducing all-cause mortality, cardiac mortality, myocardial re-infarction, and cardiovascular risk factors in high and low and middle-income countries (British Association for Cardiovascular Prevention and Rehabilitation [BACPR], 2017; Turk-Adawi & Grace, 2015). However, providing a center-based CR is particularly difficult in South East Asia including Sri Lanka due to the lack of human resources, space and equipment, and financial support (Ruano-Ravina et al., 2016; Turk-Adawi et al., 2019).

CHD patients have different health needs and challenges such as knowing the healthy diet, physical activity, and cardiac medications, uncertainty of future, communication, self-care, following a healthy lifestyle, and psychological and social support (Blair, Volpe, & Aggarwal, 2014). CHD patients perceived health professionals are the primary and preferred sources of information and the information needs to be provided based on their health status

and preferences (Vosbergen et al., 2013). CHD patients perceived that they are much unaware of the secondary prevention of CHD especially the need for adequate exercise and a healthy diet (Athbi & Hassan, 2019).

Physical activity

Physical activity is considered any body movement which requires energy expenditure (WHO, 2018). AHA and the American College of Cardiology Foundation (ACCF) recognized that lifestyle modification including physical activity is the class I recommendation for secondary prevention and risk reduction therapy for patients with CHD (Smith et al., 2011). Further, AHA and ACCF recommended the physical activity for CHD patients as moderate-intensity aerobic activity, such as brisk walking, at least 30 minutes, at least 5 days per week (Smith et al., 2011). Also, AHA and ACCF encouraged the patients to increase in daily lifestyle activities such as gardening and household works to improve their cardiorespiratory fitness, bring them physically active, and reduce their potential risk. A Cochrane systematic review and meta-analysis showed that exercise-based CR reduced cardiovascular mortality, hospital admission, and improvement in one or more domains in health-related quality of life (HRQOL) (Anderson et al., 2016).

Even though physical activity enhances health outcomes, many people with CHD do not engage in adequate physical activity in both developed and developing countries. The prevalence of being inadequately physically active among CHD patients in the United States of America (USA), England, and Sri Lanka were 83%, 76%, and 56.7% respectively (British Heart Foundation [BHF], 2017; Matthias, de Silva, Indrakumar, & Gunatilake, 2018; Tang, Patao, Chuang, & Wong, 2013).

Previous studies showed that low motivation and low mood to exercise, negative perception of health after the cardiac event, feeling physical restriction or fearful about exercise, and lack of knowledge on exercise hinder the CHD patients to engage in physical activity (Rogerson, Murphy, Bird, & Morris, 2012). On the other hand, adequate social support, having a reason for exercise, perceived psychological benefits of exercise, and using psychological strategies are the facilitators of physical activity (Rogerson et al., 2012).

Healthy diet

A healthy diet is one of the key factors to prevent CHD and helps for a better prognosis in CHD patients (Bhupathiraju & Tucker, 2011). A longitudinal cohort study evidence that recurrent CHD events and all-cause mortality inversely associated with Mediterranean diet score (high in vegetables, fruits, whole grains, beans, nut and seeds, and olive oil), whereas the Southern dietary pattern (added fats, fried food, eggs, and egg dishes,

organ meats, processed meats, and sugar-sweetened beverages) was adversely associated with all-cause mortality (Shikany et al., 2018). The dietary pattern is closely related to personal and social values, beliefs, and attitudes and environmental factors, health systems, and social structures (Reddy & Anitha, 2015). CHD patients stated that family support, self-control, cultural values, and hope for recovery were the motivators for a healthy diet, however, food beliefs or food for socialization, poverty or free food, cultural or family values, food habits and taste, satisfying hunger were the barriers (Barolia, Petrucka, Higginbottom, Khan, & Clark, 2019).

With the increasing morbidity and mortality from CHD, especially in low and middle-income countries, secondary prevention including exercise-based CR plays an important role to improve the prognosis of CHD patients (Anderson et al., 2016; Zengin et al., 2015). High prevalence of physical inactivity, unhealthy dietary practices, poor control of blood glucose, blood pressure (BP), blood lipid, and body weight (BW) was found among CHD patients in the world as well as Sri Lanka (Cooney et al., 2013; Kotseva et al., 2016; Matthias, de Silva, Indrakumar, & Gunatilake, 2018; Perera & Samarawickrama, 2017). Therefore, it is important to design and implement an appropriate intervention to improve the physical activity level, exercise self-efficacy, and cardiovascular risk factors in CHD patients in Sri Lanka.

Non-pharmacological interventions for improving physical activity and cardiovascular risk factors: Findings of the literature review

A literature review was conducted on Eight English databases (MEDLINE (Ovid), EMBASE (Ovid), PsychINFO (Ovid), Ovid Nursing database, CINAHL Complete (EBSCO), Cochrane Library, Web of Science Core Collection, and Scopus) to identify the randomized control trials (RCTs) which examined the effects of the non-pharmacological intervention (such as lifestyle modification, psychological intervention) on the BW or body mass index (BMI), BP, blood lipid, and /or blood glucose of CHD patients. This review included 26 RCTs published between 2001 and 2020 and reported data of 8103 participants with mean ages ranging from 50.5±10.6 (Jayawardena, Sooriyaarachchi, Punchihewa, Lokunarangoda, & Pathirana, 2019) to 65.9±9.4 years (Zhang et al., 2017) predominantly with acute coronary syndrome (ACS).

Characteristics of the intervention

Out of the 26 included studies, 15 studies were multi-faceted and included information provision related to CHD, exercise training, and behavioral changing strategies. The contents of the multi-faceted intervention varied among the included studies and

comprised exercise training, exercise prescription, motivation, education on CHD, psychosocial support, or social support, individualized goal setting, smoking cessation, risk assessment, and feedback, risk factor management, and stress management and counseling.

Out of 15 multi-faceted intervention studies, three studies (Pluss, Karlsson, Wallen, Billing, & Held, 2008; The Vestfold Heartcare Study Group, 2003; Toobert, Glasgow, & Radcliffe, 2000) delivered education with exercise training to enhance the participants' knowledge and physical activity level, 11 studies delivered education with behavioral change strategies (Chair et al., 2011, 2013; Ding et al., 2017; Fernandez et al., 2009; Lumertz Saffi, Polanczyk, & Rabelo-Silva, 2014; Maddison et al., 2019; McHugh et al., 2001; Muñiz et al., 2010; Redfern, Briffa, Ellis, & Freedman, 2008, 2009; Vale, Jelinek, Grigg, & Newman, 2003; Wang, Jiang, He, & Koh, 2016; Zhang et al., 2017), and one study provided exercise training with behavioral change strategies (Lear et al., 2006).

Five studies (Chen et al., 2016; Chow et al., 2015; Cohen et al., 2014; Munoz et al., 2007; Zheng et al., 2019) were categorized as education, in which educational techniques and methods were applied to help the participants to gain knowledge, support for the recovery from disease, and cope with their emotion and reactions whereas four studies provided supervised exercise training as their intervention (Ades et al., 2009; Currie, Bailey, Jung, McKelvie, & MacDonald, 2015; Pal et al., 2011; Raghuram et al., 2020).

Out of four studies that provided exercise training, two studies provided moderate-intensity exercise for six to 12 months (Pal et al., 2011; Raghuram et al., 2020), Currie et al (2015) study provided moderate or high-intensity exercise on the cycle ergometer for six months, the lower-intensity exercise preferred as walking for five months provided in Ades et al (2009) study. The intervention of included studies was categorized into exercise training, exercise training with behavioral change strategies, exercise training with education, education, education with behavioral change strategies, and dietary management.

Effectiveness of the intervention

Exercise training. The weekly face-to-face supervised exercise training for 30-40 minutes with low to moderate-intensity for five to 12 months had significant improvement on BMI (Hedges'g =0.3), BW (Hedges'g =0.4), PA (Hedges'g =0.2), SBP (Hedges'g =0.5), DBP (Hedges'g =0.4), TC (Hedges'g =0.4), HDL (Hedges'g =0.8), TG (Hedges'g =0.9) and LDL (Hedges'g =0.3) of CHD patients at 6 months (p<0.05), however, the significant improvement only sustained on TG (Hedges'g =0.3), HDL (Hedges'g =0.3), BMI (Hedges'g =0.3) at 12 months (Ades et al., 2009; Pal et al., 2011; Raghuram et al., 2020).

Exercise training with behavioral change strategies. Supervised exercise training with behavioral change strategies through face-to-face followed by telephone calls had significant improvement on SBP (Hedges'g =0.3)of CHD patients at 48 months ($p<0.05$) (Lear et al., 2006).

Exercise training with education. Exercise training with education on different aspects of CHD including nature, risk factors, sign and symptoms of CHD via face-to-face had significant improvement on BMI (Hedges'g =0.2), and PA (Hedges'g =0.5) of CHD patients at 24 months ($p<0.05$) (Toobert et al., 2000).

Education. The contents of the education varied among the included studies and comprised of risk factors, signs, and symptoms of CHD, dietary advice, advice to stop smoking and alcohol consumption, stress management, and benefits of exercise. Out of five studies that provided education on different aspects, the study provided education through four weekly personalized text messages for six months showed significant effects on BMI (Hedges'g =0.6), PA (Hedges'g =0.3), SBP (Hedges'g =0.6), DBP (Hedges'g =0.3), TC (Hedges'g =0.3), TG (Hedges'g =0.3) and LDL (Hedges'g =0.2) of CHD patients at 6 months ($p<0.05$) (Chow et al., 2015), however, other studies did not show any significant improvement on the outcomes.

Education with behavioral change strategies. Out of 13 studies, four studies had significant improvement on BW, BMI, LDL, TC, TG, SBP, DBP, and PA of CHD patients (Lumertz Saffi et al., 2014; McHugh et al., 2001; Redfern et al., 2008, 2009; Zhang et al., 2017). Individual goal setting and feedback evaluation, stress management, coping strategies, and health education through monthly telephone calls for 12 months had significant improvement on LDL (Hedges'g =0.08), BMI (Hedges'g =0.07), SBP (Hedges'g =0.3), DBP (Hedges'g =0.3), and BW (Hedges'g =0.1) at 12 months (Lumertz Saffi et al., 2014).

Education with behavioral change strageries such as individual assessment, self-monitoring, counseling on self-care and coping for 30 minutes through face-to-face at participants home in every two weeks for seven months underpinning by the combination of the Omaha system and Pender's health promotion model showed significant improvement on TG (Hedges'g =0.5), LDL (Hedges'g =0.4), TC (Hedges'g =0.8), BMI (Hedges'g =0.6), SBP (Hedges'g =1.0), DBP (Hedges'g =0.7), FBG (Hedges'g =0.4) in CHD patients at 7 months ($p<0.05$) (Zhang et al., 2017) whereas individualized case management approach with goal setting, individual risk assessment, self-monitoring and feedback evaluation delivered through a one-hour face-to-face consultation and three 10 minutes telephone calls for three months had significant improvement on LDL (Hedges'g =0.6), TC (Hedges'g =0.8), BMI

(Hedges'g =0.4), SBP (Hedges'g =0.6), DBP (Hedges'g =0.4), and PA (Hedges'g =0.6), in CHD patients at three months (p<0.05) (Redfern et al., 2008), however, greater significant improvement was observed on PA (Hedges'g =0.8) with consistent improvement on other outcomes at 12 months (Redfern et al., 2009).

However, none of the interventions except exercise training showed significant improvement in HDL in CHD patients.

Dietary management. Out of two studies (Frost, Brynes, Bovill-Taylor, & Dornhorst, 2004; Jayawardena et al., 2019) that examined the dietary intervention, Jayawardena et al (2019) study only showed significant improvement in BW (Hedges'g =0.03) and BMI (Hedges'g =0.003) in CHD patients at three months (p<0.05).

The total duration of the intervention in the included studies ranged from one month (Wang et al., 2016) to 48 months (Lear et al., 2006). This review suggested that a three-month multi-faceted intervention including supervised exercise training, behavioral changing strategies such as risk assessment and feedback, individualized goal-setting, self-monitoring, risk factor management, motivation, and education on the different aspects of CHD could improve the cardiovascular risk factors and physical activity level of CHD patients. To initiate the exercise behavior, this review suggested using the face-to-face approach to train the participant to engage in physical activity and this can be changed to telephone follow-up later. Furthermore, to deliver the education components of the intervention, the use of telephone calls should be considered in addition to traditional face-to-face interaction since this approach results in increased continuous support and improves physical activity and cardiovascular risk factors (Redfern et al., 2008, 2009).

Since CHD is the leading cause of cardiac-associated morbidity and mortality globally, the management of modifiable risk factors is a vital part of secondary prevention. Intervention consist of exercise training, education, and behavioral change strategies that can have beneficial effects on improving physical activity level, and cardiovascular risk factors among CHD patients.

Limitations

This review only included studies published in English. The findings of this review should be interpreted with caution since poor methodological quality including failure to blind outcomes assessors and perform allocation concealment has been observed among included studies. The limited number of studies was conducted in the developing countries, lack of theoretical underpinning for intervention, inconsistency in the mode of delivery, duration, number of sessions, frequency of exercise and education of the included studies

warrant further research to establish the effectiveness of the intervention that comprises of exercise training and education in the CHD population.

The direct application of these interventions to Sri Lanka may be challenging since most studies were conducted in western countries where the culture, beliefs, and attitudes towards CHD risk factors, management, and prevention may different from South Asian (Gany, Gill, Ahmed, Acharya, & Leng, 2013). Therefore, developing a culturally specific intervention considering the methodological pitfalls is essential to improve the cardiovascular risk factors and physical activity level in CHD population in Sri Lanka.

Methodology

Study aim and objectives

This study aims to develop and examine a culturally specific motivated, action-based intervention for improving physical activity level, exercise self-efficacy, and cardiovascular risk factors of CHD patients in Sri Lanka.

The objectives of the study as follows:-

i. To develop a culturally specific motivated, action-based intervention for improving physical activity level, exercise self-efficacy, and cardiovascular risk factors of CHD patients.
ii. To examine the effectiveness of a motivated, action-based intervention on improving physical activity level, exercise self-efficacy, and cardiovascular risk factors of CHD patients.

Research hypothesis

1. Participants in the intervention group will have a significantly higher level of physical activity compared with participants in the control group at immediate and one-month post-intervention.
2. Participants in the intervention group will have a significantly higher level of exercise self-efficacy compared with participants in the control group at immediate and one-month post-intervention.
3. Participants in the intervention group will have significantly improved cardiovascular risk factors namely BW, BMI, WHR, BP, lipid levels, and FBG compared with participants in the control group at immediately after the intervention.
4. Participants in the intervention group will have significantly improved cardiovascular risk factors namely BW, BMI, WHR, BP, and FBG compared with participants in the control group at one-month post-intervention.

Version – 2

Study design

An assessor-blinded randomized control trial will be conducted to assess the effectiveness of the motivated action-based intervention on the selected outcomes. The participants will be patients who are admitted to the coronary care unit (CCU) and medical wards of the Teaching Hospital Batticaloa, Sri Lanka with a diagnose of CHD at first instance. A thorough explanation of the study by using the information sheet (Appendix A) will be provided to all eligible participants and informed consent also will be obtained from the participants before the commencement of the study (Appendix B).

Study setting and subjects

The study protocol and implementation will have complied with the principles of Declaration of Helsinki (World Medical Association, 2013). Firstly, ethical approval will be obtained from The Joint Chinese University of Hong Kong – New Territories East Cluster Clinical Research Ethics Committee (The Joint CUHK-NTEC CREC) and the Ethics Review Committee, Faculty of Health Care-sciences, Eastern University, Sri Lanka. Permission also will be obtained from the director of the Teaching hospital Batticaloa, Sri Lanka. Secondly, the purpose of the study, the data collection procedures, the potential risk and benefits, the maintenance of confidentiality, and the voluntary basis of participation will be clearly explained to the participants. Participants will be informed that they have the choice to withdraw from the study at any time if they choose to do without penalty or negative consequence of current health services.

Thirdly, the informed consent will be provided in written and oral form when the research personnel approaches the potential participants. The detailed information and explanation of the nature of the study, the right of participants to withdraw from the study at any time, the anonymity and confidentiality of the participants, contact details of the researcher which participants may contact to obtain further information if they need will be included in the subject information sheet in both English and Tamil version. Fourthly, to maintain the anonymity of the participants, they will be asked not to write their name in the questionnaire, however, the code number will be provided by the researcher. All data will be kept by the principal investigator in a password-protected computer and a locked cabinet to ensure the confidentiality of participants. The participants will be informed that all personal data will only be accessed by the principal investigator and all the collected data will be destroyed five years after the study completion.

This study will be conducted in the Teaching Hospital Batticaloa, Sri Lanka. The study site is a hospital with 1200 beds that serves a population of more than two million in

the Eastern province and adjacent districts in Sri Lanka. The majority of the CHD patients of the eastern part of Sri Lanka will be admitted to this hospital for medical treatment. Study participants will be recruited from the coronary care unit and medical wards of the study hospital. The intervention will be delivered at the cardiology clinic of the hospital. The clinic provides a multidisciplinary service, including cardiologists, physicians, nurses, and other health care workers. Cardiac patients attending other OPD services are also referred to as the cardiac clinic for health assessment and unstructured health education.

Inclusion and exclusion criteria

The eligibilities of participants are; adults aged 18 years or above; patients admitted to hospital with CHD for the first time confirmed by electrocardiogram, able to reads and speak Tamil; able to attend clinic follow-up, and obtain a medical clearance from a cardiologist to perform the exercise. The medical records of the CHD patients will be reviewed to screen for their eligibility. In addition, the cardiologist of the participants will be consulted for their suitability to perform the exercise of the intervention.

The exclusion criteria are patients with absolute and relative contradictions to perform the exercise or at high risk for exercise, namely rest ejection fraction less than 40%, history of cardiac arrest or sudden death, complex dysrhythmias at rest or during exercise testing or recovery, complicated myocardial infarction or revascularization procedure, high level of silent ischemia during exercise testing or recovery, presence of abnormal hemodynamics with exercise testing or recovery and presence of angina or other significant symptoms according to the American College of Sports Medicine (ACSM) guideline and American Association of Cardiovascular and Pulmonary Rehabilitation (AACVPR) guideline (American College of Sports Medicine [ACSM], 2014); any diagnosis of life-threatening conditions or psychotic disease or unable to perform physical activity independently.

Randomization:- A stratified randomization by the sex of the participants will be conducted since sex influences the level of physical activity among CHD patients (Jason, McGannon, Blanchard, Rainham, & Dechman, 2014). The block randomization with a block size of eight will be used for group allocation of participants either to an intervention or control group with a ratio of 1: 1. The random sequencing for group allocation will be generated by an online application (http://www.randmization.com). The group allocation will be put into an opaque sealed envelope by a person who is independent of the study. After the baseline assessment (T_0), the opaque sealed envelope will be distributed to the participants according to their sequence of entry.

Version – 2

Sample size estimation

The sample size estimation is based on the primary outcomes of the study, namely the physical activity level. Referring to the finding of the litearture review, the effect size on improving physical activity is 0.5 (Redfern et al., 2008) to 0.8 (Redfern et al., 2009). Therefore, the smallest effect size of 0.5 (Redfern et al., 2008) will be considered to calculate the sample size for this study. To achieve a power of 1-β=0.8 with a significance level of 0.05 (two sides) and account for 14% of attrition rate (Maddison et al., 2019) of the previous study. A total of 150 participants will be recruited in this study.

Development of the motivated, action-based intervention

The motivated, action-based intervention for this study is developed based on the findings of the literature review and the health action process approach (HAPA) for behavioral changes (Schwarzer, 2008). Health behaviour change is fundamental to cardiovascular disease prevention and rehabilitation (BACPR, 2017). A conceptual model that could help to explain behavioral changes is needed for developing an evidence-based intervention to improve the health outcomes of CHD patients.

Self-efficacy refers to an individual’s judgment on their ability to perform the assigned action to deal with a prospective situation (Bandura, 1982) and it is a key determinant of health behavior since it, directly and indirectly, influencing health behaviors (Bandura, 2004). Further, it is one of the focus determinants of physical activity, and a higher level of self-efficacy positively correlates with physical activity (Bauman et al., 2012). The HAPA constructs assess the self-efficacy in the different stages to initiate and maintain the desired health behavior and fill the intention-behavior gap which is the key part of the behavioral change intervention (Schwarzer, 2008). Therefore, HAPA is used as a conceptual framework to initiate and maintain the behavioral changes (physical activity) in CHD population.

Health action process approach (HAPA)

The HAPA is a social-cognitive model specifying motivational and volitional determinants of health behavioral (Schwarzer, 2008) and was used among health education and promotion interventions such as improving physical activity (Lippke, Schwarzer, Ziegelmann, Scholz, & Schüz, 2010), and dietary behaviors (Wiedemann et al, 2009). HAPA comprises two phases namely, the motivation phase and the volition phase which emphasis the initiation, and maintenance of health behaviors. Individuals choose what to do in the motivation phase, whereas they try to act and maintain the formed behavior in the volitional phase.

Motivational phase

The motivational phase is the initial part of the HAPA framework in which the individual chooses what to do for changing their behavior. The intention is the initial step in the motivational phase to change individual health behavior and can be developed by enhancing risk perception, improving outcomes expectancies, and increasing the action self-efficacy of an individual (Schwarzer, 2008). Risk perception refers to individual beliefs on their health problems experienced and higher perceived risk on disease experienced could motivate the individuals to adopt a health-protective behavior. The outcome expectancies refer to an individual's belief that a specific behavior will lead to desired outcomes and it could enhance the individual's action self-efficacy. Action self-efficacy refers to an individual's judgment on their ability to perform the assigned action and can be developed or increased by performance accomplishments, social modeling, improving physical and emotional status, and verbal persuasion (Rajati et al., 2014). Good planning helps the formed intention in the motivation phase (goal-setting phase) translates into action in the volition phase (goal pursuit phase) (Zarski et al, 2018).

Volition phase

To result in behavior change, the developed intention has to be taken into action. The individual capability to deal with barriers that arise during behavioral action is called maintenance self-efficacy (Luszczynska, & Schwarzer, 2003) and it helps the individual to plan the action. The formed intention could be transformed into the proposed action through planning. The individual has to plan when, where, and how to act on the formed intention (action planning) and developed coping strategies to deal with anticipating difficulties or barriers (Coping planning) that might hinder the implementation of one's behavioral intention (Sniehotta, Scholz, & Schwarzer, 2005). Recovery self-efficacy refers to one's confidence in being capable of resuming a difficult behavior after an interruption and help to continue the achieved desired behavior (Schwarzer, 2008).

Study intervention

Participants in the intervention group will receive the motivated action-based intervention in addition to the usual care. Meanwhile, those who are in the control group will only receive the usual care provided by the cardiology clinic of the study hospital. The motivated action-based intervention and usual care will be described as follows.

Control group

The control group will receive the usual health care services for CHD patients provided in the study hospital included an unstructured health education conducted by a nurse

on a healthy lifestyle (exercise, heart-healthy diet, stress management, and smoking cessation) will be given once a month for one year after discharge through face-to-face. During the first visit of CHD patient to the cardiology clinic two weeks after discharge, the cardiologist assesses the patient's condition and giving brief unstructured health education on their conditions, focusing on the risk factors management and stress management for 10-15 minutes. The patients are also instructed to visit the cardiology clinic every month for medical follow-up.

Motivated action-based intervention (Intervention Group)

Based on the findings of the literature review, the essential components of the intervention to improve cardiovascular outcomes included individual risk assessment and feedback, individualized goals setting and an action plan, supervised exercise training, education on lifestyle modification, nature, risk factors, sign and symptoms, medication management and adherence for CHD. To initiate and maintain these behavioral changes of CHD patients, the motivated action-based intervention is developed according to the HAPA model. The motivated action-based intervention comprises two phases, namely the face-to-face preparatory phase and a 12-week intervention phase. The intervention will be delivered by a researcher who has experience in clinical teaching and patient education.

Face-to-face preparatory phase

The preparatory phase includes one individualized health counseling for 60 minutes will be conducted by the researcher at the cardiology clinic at the hospital within three months after the CHD event. During this session, the participant's health needs will be recognized by assessing the health condition, facilitate the participants to review their daily activities and explore the discrepancy between activity level and the recommended exercise guidelines, identify the three desired outcomes (achievable and observable), form individualized exercise goals and a behaviorally specific action plan for behavioral changes. In addition, knowledge and overview of the programme and real success stories will also be shared with participants.

The Framingham risk score (FRS) is a simple and common tool use to assess the risk level of CHD over ten years (Wannamethee, Shaper, Lennon, & Morris, 2005) and its comprise of six coronary risk factors namely, age, gender, TC, HDL, SBP and smoking habits (Sohn, Kim, & Bae, 2012). Furthermore, it can be used for lifestyle modification of patients by identifying their risk level (Wilson et al., 1998). The self-reported risk of CHD will be compared with the calculated FRS to identify the discrepancy in the risk level and for enhancing the risk perception of participants.

Recognizing and addressing outcome expectancies of the patients is one of the important aspects of the behavioral change intervention to adhere to the behavior change (Reesor, Vaughan, Hernandez, & Johnston, 2017). The participants will be asked to list the outcome expectancies (positive and negative) for following a healthy lifestyle such as engaging adequate physical activity, stop smoking, eating a healthy diet, and other risk factor management and not following a healthy lifestyle. Then the researcher will discuss the validity and reality of each expectation and encourage the participants to understand the benefits of healthy behaviour and motivate them to follow healthy behaviour.

Furthermore, the participant's concerns and questions will be addressed in this phase to alleviate their fear and anxiety to perform the exercise and enhance their knowledge of CHD. Ask the participants to list their achievements and appreciation will be given to them to initiate the desired behavior. These strategies will help to initiate the action self-efficacy of HAPA.

Intervention phase

The 12-week intervention consists of three monthly group education and center-based group exercise followed by 20 minutes of individualized telephone follow-up at weeks 3, 7, and 11 will be conducted one week after the preparatory phase to transform the intention into action and to maintain the behaviour change.

Education. As evidence from the literature review, an educational package will be developed and used in the education sessions to improve the patient's knowledge of CHD risk and its management. The educational package consists of the following topics: Nature of CHD, risk factors, signs and symptoms of CHD, heart-healthy diet, physical activity, quit smoking, stress management, and medication management and adherence (American Cancer Society, 2020; AHA, 2014; American Psychological Association, 2020; Eckel et al., 2014; Price, Gordon, Bird, & Benson, 2016; Scott et al, 2018; Somasundaram et al, 2013; Somasundaram et al, 2014).

The content of the booklet will be validated by an expert panel consists of registered physiotherapists, registered nutritionists, cardiologists, and the cardiac nurse for their safety and appropriateness for CHD patients in Sri Lanka to provide culturally relevant written resources to encourage the patients to follow the health advice at home. The knowledge gained in the education will help the participants to form the intention by increasing risk perception, positive outcome expectancies, and action self-efficacy in the motivational phase of HAPA. Further, the education will also support the participants to plan the action of

intended behavior and help to maintain self-efficacy throughout the volitional phase in HAPA.

To change individual behavior, three monthly face-to-face group education will be conducted. Group education sessions consist of 10 participants will be delivered as follows at the cardiology clinic of the hospital: Nature, risk factors and signs and symptoms of CHD (30 minutes), heart-healthy diet, and physical activity (30 minutes), quit smoking, and stress management (30 minutes), and medication management and adherence (20 minutes). Further, the participants will be educated on how to complete food, medication, and exercise diaries to visualize their behavioral changes. The education booklet will be provided to the participants and telephone follow-up at weeks 3, 7, and 11 will be made. The participant's concerns and questions will be addressed during each telephone contact to enhance the maintenance self-efficacy, action planning, and coping planning of the HAPA.

Exercise. Apart from the group education, a hospital plus home-based heart-healthy exercise will be implemented during these 12 weeks to promote the behavioral change for better health outcomes. The maintenance self-efficacy from HAPA will use to help the patient to overcome the barriers during the exercise program. Music will be used during the exercise sessions to guide the participants to perform the exercise at the prescribed intensity and duration and reduces the rating of perceived exertion (RPE) among cardiac patients (Cheng, & Grove, 2017; Miller & Terbizan, 2017).

The exercise intervention comprised of two elements: (1) three monthly center-based exercise program, and (2) a 12 weeks home-based exercise program

(1) The three monthly hospital-based group exercises consists of eight to ten participants in each group will be implemented with the support of a physiotherapist, registered nurse, medical officer, and researcher. A total of three group exercise sessions once per month for three months will be conducted at the study hospital. During the first two sessions, participants will be guided to perform the exercise (warm-up exercise, brisk walking, and cool-down exercise) with the music and under the supervision of a physiotherapist of the study hospital. As suggested by the cardiologist, the exercise will start at low-intensity with cultural background music (Raravenu Gopabala Instrumental flute version) with a slow tempo (40-60 beats per minute). Before starting the walking exercise, the participants will perform the warm-up exercise for 5 minutes. Then participants will be asked to walk for 20 minutes with the speed paced by the music tempo of Raravenu Gopabala Instrumental flute version. After completing the walking, the participants will perform the cool-down exercise for 5 minutes.

A total of two exercise sessions (brisk walking), 20 minutes/session) one per month will be implemented for the first two months at the hospital. From the third month onwards, the intensity of the aerobic exercise will increase to moderate-intensity and the duration will be increased to 30 minutes/session.

(2) Twelve-week home-based exercise will be conducted between hospital-based exercise sessions. The participants will be asked to continue the exercise performed in the previous week at the hospital with culturally background music for three times per week. An exercise logbook will be distributed to the participants and the researcher will teach the participant how to fill the logbook and how to follow the exercise plan for home-based practice. This exercise log record will allow the participants to self-monitoring and will use to identify the tract in their exercise practice at home will help to maintain the action and coping planning of the HAPA.

Telephone follow-up. The researcher will provide a 20-minutes telephone follow-up at weeks 3, 7, and 11 for strengthening the volition of the participant to perform the exercise and follow the healthy diet at home and to enhance problem-solving skills to overcome perceived barriers and to provide reinforcement for any positive changes that resulted. During telephone follow-up, the patient will be instructed to go through a series of self-management steps, including an assessment on current exercise and diet practice, giving feedback on consistency or any discrepancy between the designated plan and current practice, identifying facilitators and barriers for behavioral changes, remind them to follow the educational manual and perform the prescribed exercise, exploring problem-solving strategies, and strengthening confidence and motivation. In each telephone follow-up, the goals and action plan will be reviewed and mutually adjusted as necessary for the next follow-up review.

Data collection

The study outcomes will be assessed at baseline (T_0), upon completion of the 12-weeks intervention (T_1), and one-month post-intervention (T_2) by a trained research assistant (RA) who is blinded for the group allocation. Every participant will receive 230 Sri Lankan rupees (one United States dollar) after completing data collection to compensate for the time spending on data collection. The flow chart for the main study is shown in Appendix C.

Outcome measures

The primary outcome of this study is the physical activity level of the participants while the secondary outcomes are cardiac exercise self-efficacy and cardiovascular risk factors namely, BW, BMI, WHR, BP, lipids levels, and FBG. Physical activity level, cardiac exercise self-efficacy, BW, BMI, WHR, BP, and FBG will be measured at baseline, at

immediate post-intervention and on-month post-intervention, whereas lipids profile will be measured at baseline and immediate post-intervention.

Informed written consent will be obtained from the participants before the study commencement. The interview will be conducted to collect the information by a trained RA.

Socio-demographic and clinical data form: The socio-demographic characteristics of the participants, including their gender, age, marital status, ethnicity, education level, employment, and family monthly income, will be collected. Furthermore, the clinical characteristics of the participants, including the duration of illness, the medication history, and other comorbidities will be collected (Appendix D).

Cardiac Exercise Self-Efficacy Instrument (CESEI): The CESEI is a 16-item instrument developed to assess the individual's confidence to perform the physical exercise in cardiac patients (Hickey, Owen, & Froman, 1992). Each item is rated on a five-point Likert scale from 1 (very little confidence) to 5 (quite a lot of confidence). Participants will be asked to rate their confidence level in performing exercise in various situations such as "fitting exercise into a busy day," "exercising without getting chest pain," "taking own heart rate before and after exercise," and "enduring light exercise". The CESEI score range from 16-80 and a higher score indicates that a high level of self-efficacy in performing the exercise. The CESEI demonstrated high reliability with high internal consistency (Cronbach's alpha = 0.9) and test-retest reliability (r = 0.86) and known-group construct validity in exercise self-efficacy between marathon runners and cardiac rehabilitation patients ($p = 0.04$) (Hickey et al., 1992). The exercise self-efficacy level of participants will be measured using a validated Tamil version of CESEI (Appendix E).

International Physical Activity Questionnaire Short Form (IPAQ-SF): IPAQ-SF questionnaire consists of 7 items to assess the physical activity of an individual with various intensities (Craig et al., 2003). The first six questions of IPAQ-SF focus on three specific types of activity in the last seven days, namely walking, moderate-intensity activities, and vigorous-intensity activities. The final question asks about the time "spent sitting" on weekdays. Furthermore, the original version of IPAQ-SF demonstrated high reliability with test-retest reliability (Craig et al., 2003). The validated Tamil version of IPAQ-SF will be used to assess the self-reported time spent in walking, moderate and vigorous-intensity activities, and sitting in this study (Appendix F).

All anthropometric measurements will be performed by a trained research assistant who will be blinded to the group allocation of participants. The weight will be measured with a calibrated mechanical personal scale (model-CAMRY, model number-BS 20140).

Participants will be weighed wearing light clothes, without shoes, and an empty bladder. The height will be measured in a standing position by using a plastic flexible tape (without shoes) and BMI will be calculated as weight in kilograms divided by height squared in meters (kg/m^2). WC will be measured using a non-stretchable measuring tape at the approximate midpoint between the lower margin of the last palpable rib and the top of the iliac crest to the nearest 1cm, at end of the normal expiration. Hip circumference will be measured at the widest level over the grater trochanters using plastic flexible tape to the nearest 0.1 cm. WHR will be estimated as waist measurement (cm) divided by hip measurement (cm).

BP in a sitting position will be measured using a mercury sphygmomanometer (Dekamet model Code: 0125). Participants will be asked to rest for 15 minutes and no caffeinated food or drink 30 minutes before the BP measurement. The participants' lipid profiles and FBG will be obtained from their clinical records at baseline and immediate post-intervention.

Data analysis

Data will be analysed using SPSS version 22.0 statistical software with a 5% significance level (two-sided) (SPSSinc., Chicago, II, USA). Data will be analysed using descriptive and inferential statistics. Descriptive statistics with mean, standard deviation, range, frequency, and the percentage will be used to describe the participants' socio-demographic characteristics and clinical data. The normality of the data will be tested using skewness and kurtosis statistics and graphically by the normal Q-Q plots. While an independent t-test will be used to compare the baseline characteristics of the intervention and control group for continuous variables, the Chi-square test will be used for categorical variables. The generalized estimating equation (GEE) model will be used for hypotheses testing, to determine significant differences across different time points (effect of time, group, and time x group interaction). The intention-to-treat (ITT) analysis will be used as an approach to analyse all participants in the final analysis according to the original assigned group irrespective of treatment received. If these assumptions are not met, the equivalent non-parametric statistics will be used to analyse the data.

Ethical consideration

Ethical approval will be obtained from The Joint Chinese University of Hong Kong – New Territories East Cluster Clinical Research Ethics Committee (The Joint CUHK-NTEC CREC) and Ethics Review Committee, Faculty of Health Care-sciences, Eastern University, Sri Lanka. Permission will also be obtained from the director of Teaching Hospital, Batticaloa, Sri Lanka. The study will adhere to the Declaration of Helsinki (World Medical

Association, 2013). Written informed consent of the participants will be obtained before data collection. Participants' safety in this intervention will be ensured as follows: the medical clearance to participate in this intervention to all eligible participants will be obtained from their cardiologist before participating in the intervention, the brisk walking is a safe and simplest exercise for improving cardiovascular risk factors (Centre for health protection, Department of health, 2020), the exercise sessions will be delivered as supervised brisk walking, and the contact details of the cardiologist and researcher will be provided to all participants to inform if patients develop any discomforts. The purpose of the study, the data collection procedures, the potential risk and benefits, the maintenance of confidentiality, and the voluntary basis of participation will be clearly explained to the participants. The right of participants to withdraw from the study at any time without providing a reason and their refusal to participate and withdraw from the study would not affect the amount of care they receive from the hospital will be reinforced. The name of the participants will be coded, and the code will be stored separately to ensure anonymity. All data will be kept by the principal investigator in a password-protected computer and a locked cabinet. The personal data will only be accessed by the principal investigator. All the collected data will be destroyed five years after the study completion.

Significance of the study

This study will provide evidence on the effectiveness of a motivated, action-based intervention on the physical activity level, cardiovascular risk factors, and exercise self-efficacy of CHD patients in Sri Lanka. Findings from this study could be useful to promote healthy lifestyle behaviors in CHD patients in a low resource setting.

Reference list

Ades, P. A., Savage, P. D., Toth, M. J., Harvey-berino, J., Schneider, D. J., Bunn, J. Y., … Ludlow, M. (2009). High-caloric expenditure exercise: A new approach to cardiac rehabilitation for overweight coronary patients Ades: High- caloric exercise overweight coronary patients. *Circulation*, *119*(20), 2671–2678. http://doi.org/10.1161/CIRCULATIONAHA.108.834184.High-Caloric

Alhabib, K. F., Sulaiman, K., Al-motarreb, A., Almahmeed, W., Asaad, N., Amin, H., … Heart, T. G. (2020). Baseline characteristics, management practices, and long-term outcomes of Middle Eastern patients in the second gulf registry of acute coronary events (Gulf RACE-2). *Ann Saudi Med*, *32*(2), 9–18. http://doi.org/10.5144/0256-4947.2012.9

American Cancer Society. (2020). How to quit smoking or smokeless tobacco. Retrieved from https://www.cancer.org/healthy/stay-away-from-tobacco/guide-quitting-smoking.html

American College of Sports Medicine. (2014). *ACSM'S Guidelines for Exercise Testing and Prescription.* Retrieved from www.acsm.org

American Heart Association. (2014). 3 Tips to manage stress. Retrieved from https://www.heart.org/en/healthy-living/healthy-lifestyle/stress-management/3-tips-to-manage-stress

American Heart Association. (2015). Coronary artery disease - Coronary heart disease. Retrieved from https://www.heart.org/en/health-topics/consumer-healthcare/what-is-cardiovascular-disease/coronary-artery-disease

American Psychological Association. (2020). Psychology help center. Retrieved from https://www.apa.org/helpcenter/index

Anderson, L., Oldridge, N., Thompson, D. R., Ann-Dorthe Zwisler, Rees, K., Martin, N., & Rod S. Taylor. (2016). Exercise-based cardiac rehabilitation for coronary heart disease: Cochrane systematic review and meta-analysis. *Journal of the American College of Cardiology*, *67*(1), 1–12. http://doi.org/10.1016/j.jacc.2015.10.044

Athbi, H. A., & Hassan, H. B. (2019). Health beliefs of patients with coronary heart disease toward secondary health beliefs of patients with coronary heart disease toward secondary prevention : The health beliefs model as a theoretical framework. *Indian Journal of Forensic Medicine & Toxicology*, *13*(2), 273–278. http://doi.org/10.5958/0976-5506.2019.00161.X

Bandura, A. (1982). Self-efficacy mechanism in human agency. *American Psychologist*, *37*(2), 122–147.

Bandura, A. (2004). Health promotion by social cognitive means. *Health Education and Behavior*, *31*(2), 143–164. http://doi.org/10.1177/1090198104263660

Barolia, R., Petrucka, P., Higginbottom, G. A., Khan, F. F. S., & Clark, A. M. (2019). Motivators and deterrents to diet change in low socio-economic Pakistani patients with cardiovascular disease. *Global Qualitative Nursing Research*, *6*, 1–10. http://doi.org/10.1177/2333393619883605

Bauman, A. E., Reis, R. S., Sallis, J. F., Wells, J. C., Loos, R. J. F., & Martin, B. W. (2012). Correlates of physical activity: Why are some people physically active and others not? *The Lancet*, *380*(9838), 258–271. http://doi.org/10.1016/S0140-6736(12)60735-1

Bhupathiraju, S. N., & Tucker, K. L. (2011). Coronary heart disease prevention: Nutrients, foods, and dietary patterns. *Clin Chim Acta*, *412*(17–18), 1493–1514. http://doi.org/10.1016/j.cca.2011.04.038.Coronary

Blair, J., Volpe, M., & Aggarwal, B. (2014). Challenges, needs, and experiences of recently hospitalized cardiac patients and their informal caregivers. *J Cardiovasc Nurs*, *29*(1), 1–15. http://doi.org/10.1097/JCN.0b013e3182784123.Challenges

British Association for Cardiovascular Prevention and Rehabilitation. (2017). *The BACPR*

standards and core competencies for cardiovascular disease prevention and rehabilitation (3rd ed., Vol. 2017). London: BACPR. Retrieved from http://www.bacpr.com/resources/6A7_BACR_Standards_and_Core_Components_2017.pdf

British Heart Foundation. (2017). *Physical inactivity and sedentary behaviour report 2017*. Retrieved from https://www.bhf.org.uk/informationsupport/publications/statistics/physical-inactivity-report-2017

Centre for health protection, Department of health, The government of the Hong Kong special administrative region. (2020). Exercise and health - Brisk walking. Retrieved from https://www.chp.gov.hk/en/static/90005.html

Chair, S. Y., Chan, S. W.-C., Thompson, D. R., Leung, K.-P., Ng, S. K.-C., & Choi, K. C. (2011). Short-term effect of motivational interviewing on clinical and psychological outcomes and health-related quality of life in cardiac rehabilitation patients with poor motivation in Hong Kong: A randomized controlled trial. *European Journal of Preventive Cardiology*, *19*(6), 1383–1392. http://doi.org/10.1177/1741826711425428

Chair, S. Y., Chan, S. W.-C., Thompson, D. R., Leung, K.-P., Ng, S. K.-C., & Choi, K. C. (2013). Long-term effect of motivational interviewing on clinical and psychological outcomes and health-related quality of life in cardiac rehabilitation patients with poor motivation in Hong Kong : A randomized controlled trial. *Clinical Rehabilitation*, *27*(12), 1107–1117. http://doi.org/10.1177/0269215513490527

Chen, J., Lin, T., Voon, W., Lai, W., Huang, M.-H., Sheu, S.-H., & Chen, C.-K. (2016). Beneficial effects of home-based cardiac rehabilitation on metabolic profiles in coronary heart-disease patients. *Kaohsiung Journal of Medical Sciences*, *32*, 267–275. http://doi.org/10.1016/j.kjms.2016.04.014

Cheng M.A, Grove, T. (2017). Classical music in cardiac prevention and rehabilitation. *British Journal of Cardiac Nursing*, *12*(12), 598–609. Retrieved from magonlinelibrary.com

Chow, C. K., Redfern, J., Hillis, G. S., Thakkar, J., Santo, K., Hackett, M. L., … Thiagalingam, A. (2015). Effect of lifestyle-focused text messaging on risk factor modification in patients with coronary heart disease: A randomized clinical trial. *JAMA*, *314*(12), 1255–1263. http://doi.org/10.1001/jama.2015.10945

Cohen, A., Assyag, P., Boyer-Chatenet, L., Cohen-Solal, A., Perdrix, C., MarieDalichampt, … Boutron, I. (2014). An education program for risk factor management after an acute coronary syndrome: A randomized clinical trial. *JAMA Intern Med*, *174*(1), 40–48. http://doi.org/10.1001/jamainternmed.2013.11342

Cooney, M. T., Kotseva, K., Dudina, A., Backer, G. De, Wood, D., & Graham, I. (2013). Determinants of risk factor control in subjects with coronary heart disease: a report from the EUROASPIRE III investigators. *European Journal of Preventive Cardiology*, *20*(4), 686–691. http://doi.org/10.1177/2047487312445562

Craig, C. L., Marshall, A. L., Sjöström, M., Bauman, A. E., Booth, M. L., Ainsworth, B. E., … Oja, P. (2003). International physical activity questionnaire: 12-country reliability and validity. *Medicine and Science in Sports and Exercise*, *35*(8), 1381–1395. http://doi.org/10.1249/01.MSS.0000078924.61453.FB

Currie, K. D., Bailey, K. J., Jung, M. E., McKelvie, R. S., & MacDonald, M. J. (2015). Effects of resistance training combined with moderate-intensity endurance or low-volume high-intensity interval exercise on cardiovascular risk factors in patients with coronary artery disease. *Journal of Science and Medicine in Sport*, *18*, 637642. http://doi.org/10.1016/j.jsams.2014.09.013

Ding, R., Li, J., Gao, L., Zhu, L., Xie, W., Wang, X., … Hu, D. (2017). The effect of home-

based cardiac rehabilitation on functional capacity, behavior, and risk factors in patients with acute coronary syndrome in China. *Cardiovascular Innovations and Applications*, *2*(2), 253–264. http://doi.org/10.15212/CVIA.2017.0004

Eckel, R. H., Jakicic, J. M., Ard, J. D., Jesus, J. M. De, Miller, N. H., Hubbard, V. S., … Shen, W. (2014). AHA/ACC prevention guideline 2013 AHA/ACC guideline on lifestyle management to reduce the cardiovascular risk a report of the American College of Cardiology/American Heart Association Task Force on practice guidelines. *Circulation*, *129*(2), 76–99. http://doi.org/10.1161/01.cir.0000437740.48606.d1

Fernandez, R. S., Davidson, P., Griffiths, R., Juergens, C., Stafford, B., & Salamonson, Y. (2009). A pilot randomized controlled trial comparing a health-related lifestyle self-management intervention with standard cardiac rehabilitation following an acute cardiac event: Implications for a larger clinical trial. *Australian Critical Care*, *22*, 17–27. http://doi.org/https://dx.doi.org/10.1016/j.aucc.2008.10.003

Frost, G. S., Brynes, A. E., Bovill-Taylor, C., & Dornhorst, A. (2004). A prospective randomized trial to determine the efficacy of a low glycaemic index diet given in addition to healthy eating and weight loss advice in patients with coronary heart disease. *European Journal of Clinical Nutrition*, *58*, 121–127. http://doi.org/10.1038/sj.ejcn.1601758

Gany, F. M., Gill, P. P., Ahmed, A., Acharya, S., & Leng, J. (2013). Every disease.. man can get can start in this cab: Focus groups to identify South Asian taxi drivers' knowledge, attitudes, and beliefs about cardiovascular disease and its risks. *J Immigr Minor Health*, *15*(5), 986–992. http://doi.org/10.1007/s10903-012-9682-7.

Hickey, L. M., Owen, V. S., & Froman, D. . (1992). Instrument development: cardiac diet and exercise self-efficacy. *Nursing Research*, *41*(6), 347–353.

Jason, T., McGannon, K. R., Blanchard, C. M., Rainham, D., & Dechman, G. (2014). A systematic gender-based review of physical activity correlates in coronary heart disease patients. *International Review of Sport and Exercise Psychology*, *8*(1), 1–23. http://doi.org/10.1080/1750984X.2014.932425

Jayawardena, R., Punchihewa, P., Ranathunga, I., Lokunarangoda, N., Pathirana, A. K., & Santharaj, W. S. (2016). Body weight perception among Sri Lankan cardiac patients. *BMC Obesity*, *3*(1), 32. http://doi.org/10.1186/s40608-016-0113-5

Jayawardena, R., Sooriyaarachchi, P., Punchihewa, P., Lokunarangoda, N., & Pathirana, A. K. (2019). Effects of "plate model" as a part of dietary intervention for rehabilitation following myocardial infarction: A randomized controlled trial. *Cardiovasc Diagn Ther*, *9*(2), 179–188. http://doi.org/10.21037/cdt.2019.03.04

Lear, S. A., Spinelli, J. J., Linden, W., Brozic, A., Kiess, M., Frohlich, J. J., & Ignaszewski, A. (2006). The extensive lifestyle management intervention (ELMI) after cardiac rehabilitation: A 4-year randomized controlled trial. *Am Heart J*, *152*, 333–339. http://doi.org/10.1016/j.ahj.2005.12.023

Lippke, S., Schwarzer, R., Ziegelmann, J. P., Scholz, U., & Schüz, B. (2010). Testing stage-specific effects of a stage-matched intervention : A randomized controlled trial targeting physical exercise and its predictors. *Health Education & Behavior*, *37*(4), 533–546. http://doi.org/10.1177/1090198109359386

Lumertz Saffi, M. A., Polanczyk, C. A., & Rabelo-Silva, E. R. (2014). Lifestyle interventions reduce cardiovascular risk in patients with coronary artery disease: A randomized clinical trial. *European Journal of Cardiovascular Nursing*, *13*(5), 436–443. http://doi.org/10.1177/1474515113505396

Luszczynska, A., & Schwarzer, R. (2003). Planning and self-efficacy in the adoption and maintenance of breast self-examination: A longitudinal study on self-regulatory cognitions. *Psychology and Health*, *18*, 93–108.

http://doi.org/10.1080/08870440210000019358

Maddison, R., Rawstorn, J. C., Stewart, R. A. H., Benatar, J., Whittaker, R., Rolleston, A., … Gant, N. (2019). Effects and costs of real-time cardiac telerehabilitation: Randomised controlled non-inferiority trial. *Heart*, *105*, 122–129. http://doi.org/10.1136/heartjnl-2018-313189

Matthias, A. T., de Silva, D. K. N., Indrakumar, J., & Gunatilake, S. B. (2018). Physical activity levels of patients prior to acute coronary syndrome – Experience at a tertiary care hospital in Sri Lanka. *Indian Heart Journal*, *70*(3), 350–352. http://doi.org/10.1016/j.ihj.2017.08.020

McHugh, F., Lindsay, G. M., Hanlon, P., Hutton, I., Brown, M. R., Morrison, C., & Wheatley, D. J. (2001). Nurse-led shared care for patients on the waiting list for coronary artery bypass surgery: A randomized controlled trial. *Heart*, *86*, 317–323. Retrieved from http://ovidsp.ovid.com/ovidweb.cgi?T=JS&PAGE=reference&D=medc2&NEWS=N&AN=11514487

Miller, J. S., & Terbizan, D. J. (2017). Clinical outcomes of different tempos of music during exercise in cardiac rehabilitation patients. *International Journal of Exercise Science*, *10*(5), 681–689.

Muñiz, J., Gómez-Doblas, J. J., Santiago-Pérez, M. I., Lekuona-Goya, I., Murga-Eizagaetxebarría, N., Teresa-Galván, E. de, … Castro-Beiras, A. (2010). The effect of post-discharge educational intervention on patients in achieving objectives in modifiable risk factors six months after discharge following an episode of acute coronary syndrome, (CAM-2 Project): A randomized controlled trial. *Health and Quality of Life Outcomes*, *8*, 137–145. http://doi.org/10.1186/1477-7525-8-137

Munoz, M., Vila, J., Cabañero, M., Rebato, C., Subirana, I., Sala, J., & Marrugat, J. (2007). Efficacy of an intensive prevention program in coronary patients in primary care, a randomized clinical trial. *International Journal of Cardiology*, *118*, 312–320. http://doi.org/10.1016/j.ijcard.2006.07.015

Nowbar, A. N., Gitto, M., Howard, J. P., Francis, D. P., & Al-Lamee, R. (2019). Mortality from ischemic heart disease. *Circulation. Cardiovascular Quality and Outcomes*, *12*(6), 1–10. http://doi.org/10.1161/CIRCOUTCOMES.118.005375

Nutrition Division-Ministry of Health, Sri Lanka. (2011). Food-based dietary guidelines for Sri Lankan. Retrieved from http://203.94.76.60/departmnt/NutritionDivision.html

Pal, A., Srivastava, N., Tiwari, S., Verma, N. S., Narain, V. S., Agrawal, G. G., … Kumar, K. (2011). Effect of yogic practices on lipid profile and body fat composition in patients of coronary artery disease. *Complementary Therapies in Medicine*, *19*, 122–127. http://doi.org/http://dx.doi.org/10.1016/j.ctim.2011.05.001

Perera, A., & Samarawickrama, M. (2017). Knowledge, attitude, and practices of dietary management among ischaemic heart disease patients treated at teaching hospital Karapitiya, Galle, Sri Lanka. *IOSR Journal Of Pharmacy*, *7*(1), 58–65. Retrieved from www.iosrphr.org

Pluss, C. E., Karlsson, M. R., Wallen, N. H., Billing, E., & Held, C. (2008). Effects of an expanded cardiac rehabilitation programme in patients treated for an acute myocardial infarction or a coronary artery by-pass graft operation. *Clinical Rehabilitation*, *22*, 306–318. http://doi.org/http://dx.doi.org/10.1177/0269215507085379

Price, K. J., Gordon, B. A., Bird, S. R., & Benson, A. C. (2016). A review of guidelines for cardiac rehabilitation exercise programmes : Is there an international consensus? *European Journal of Preventive Cardiology*, *23*(16), 1715–1733. http://doi.org/10.1177/2047487316657669

Raghuram, N., Parachuri, V. R., Swarnagowri, M. V., Babu, S., Chaku, R., Kulkarni, R., …

Nagendra, H. R. (2020). Yoga-based cardiac rehabilitation after coronary artery bypass surgery: One-year results on LVEF, lipid profile, and psychological states-A randomized controlled study. *Indian Heart Journal*, *66*, 490–502. http://doi.org/10.1016/j.ihj.2014.08.007

Rajati, F., Sadeghi, M., Feizi, A., Sharifirad, G., Hasandokht, T., & Mostafavi, F. (2014). Self-efficacy strategies to improve exercise in patients with heart failure: A systematic review. *ARYA Atheroscler*, *10*(6), 319–333. Retrieved from https://www.ncbi.nlm.nih.gov/pmc/articles/PMC4354085/

Ralapanawa, U., Vithanage Ranjith Kumarasiri, P., Jayawickreme, K. P., Kumarihamy, P., Wijeratne, Y., Ekanayake, M., & Dissanayake, C. (2019). Epidemiology and risk factors of patients with types of acute coronary syndrome presenting to a tertiary care hospital in Sri Lanka. *BMC Cardiovascular Disorders*, *19*(229), 1–9. http://doi.org/10.1186/s12872-019-1217-x

Reddy, S., & Anitha, M. (2015). Culture and its influence on nutrition and oral health. *Biomedical & Pharmacology Journal*, *8*, 613–620. http://doi.org/http://dx.doi.org/10.13005/bpj/757

Redfern, J., Briffa, T., Ellis, E., & Freedman, S. B. (2008). Patient-centered modular secondary prevention following acute coronary syndrome: A randomized controlled trial. *Journal of Cardiopulmonary Rehabilitation & Prevention*, *28*(2), 107–115. http://doi.org/https://dx.doi.org/10.1097/01.HCR.0000314204.86805.13

Redfern, J., Briffa, T., Ellis, E., & Freedman, S. B. (2009). Choice of secondary prevention improves risk factors after acute coronary syndrome: 1-year follow-up of the CHOICE (Choice of health options in prevention of cardiovascular events) randomized controlled trial. *Heart*, *95*, 468–475. http://doi.org/10.1136/hrt.2008.150870

Reesor, L., Vaughan, E. M., Hernandez, D. C., & Johnston, C. A. (2017). Addressing outcomes expectancies in behavior change. *American Journal of Lifestyle Medicine*, *11*(6), 430–432. http://doi.org/10.1177/1559827617722504

Rogerson, M. C., Murphy, B. M., Bird, S., & Morris, T. (2012). “I don’t have the heart”: a qualitative study of barriers to and facilitators of physical activity for people with coronary heart disease and depressive symptoms. *International Journal of Behavioral Nutrition and Physical Activity*, *9*(140), 9. http://doi.org/10.1186/1479-5868-9-140

Ruano-Ravina, A., Pena-Gil, C., Abu-Assi, E., Raposeiras, S., van ’t Hof, A., Meindersma, E., … González-Juanatey, J. R. (2016). Participation and adherence to cardiac rehabilitation programs. A systematic review. *International Journal of Cardiology*, *223*, 436–443. http://doi.org/10.1016/j.ijcard.2016.08.120

Schwarzer, R. (2008). Modeling health behavior change : How to predict and modify the adoption and maintenance of health behaviors. *Applied Psychology: An International Review*, *57*(1), 1–29. http://doi.org/10.1111/j.1464-0597.2007.00325.x

Scott M. Grundy, Neil J. Stone, Alison L. Bailey, Craig Beam, Kim K. Birtcher, Roger S. Blumenthal, Lynne T. Braun, S. de F. (2018). Guideline on the management of blood cholesterol. *J Am Coll Cardiol*, 22. http://doi.org/10.1016/j.jacc.2018.11.003

Shikany, J. M., Safford, M. M., Bryan, J., Newby, P. K., Richman, J. S., Durant, R. W., … Judd, S. E. (2018). Dietary patterns and Mediterranean diet score and hazard of in the REGARDS Study. *J Am Heart Assoc*, *7:e008078*, 1–11. http://doi.org/10.1161/JAHA.117.008078

Smith, S. C., Benjamin, E. J., Bonow, R. O., Braun, L. T., Creager, M. A., Franklin, B. A., … Taubert, K. A. (2011). AHA/ACCF guideline AHA/ACCF secondary prevention and risk reduction therapy for patients with coronary and other atherosclerotic vascular disease : 2011 Update. *Circulation*, *124*, 2458–2473. http://doi.org/10.1161/CIR.0b013e318235eb4d

Sniehotta, F. F., Scholz, U., & Schwarzer, R. (2005). Bridging the intention-behavior gap : Planning, self-efficacy, and action control in the adoption and maintenance of physical exercise. *Psychology and Health*, *20*(2), 143–160. http://doi.org/10.1080/08870440512331317670

Sohn, C., Kim, J., & Bae, W. (2012). The Framingham risk score, diet, and inflammatory markers in Korean men with metabolic syndrome. *Nutrition Research and Practice*, *6*(3), 246–253. http://doi.org/10.4162/nrp.2012.6.3.246

Somasundaram, N. P., Wijeyaratne, P. C. N., Silva, P. S. De, Siribaddana, P. S., & Illangasekera, P. U. (2013). Clinical guidelines: The endocrine society of Sri Lanka: Diabetes mellitus : Glucose control. *Sri Lanka Journal of Diabetes, Endocrinology and Metabolism*, *3*(1), 45–57.

Somasundaram N, Rajaratnam H, Wijeyarathne C, Katulanda P, De Silva S, Wickramasinghe P, et al. (2014). Clinical guidelines: The endocrine society of Sri Lanka; Management of obesity. *Sri Lanka Journal of Diabetes, Endocrinology and Metabolism*, *4*(2), 55–70.

Tang, L., Patao, C., Chuang, J., & Wong, N. D. (2013). Cardiovascular risk factor control and adherence to recommended lifestyle and medical therapies in persons with coronary heart disease (from the national health and nutrition examination survey 2007-2010). *The American Journal of Cardiology*, *112*(8), 1126–1132. http://doi.org/10.1016/j.amjcard.2013.05.064

The Vestfold Heartcare Study Group. (2003). Influence on lifestyle measures and five-year coronary risk by a comprehensive lifestyle intervention programme in patients with coronary heart disease. *Eur J Cardiovasc Prevention Rehab*, *10*, 429437. http://doi.org/10.1097/01.hjr.0000107024.38316.6a

Toobert, D. J., Glasgow, R. E., & Radcliffe, J. L. (2000). Physiologic and related behavioral outcomes from the women â€™ s lifestyle heart trial. *Ann Behav Med*, *22*(1), 1–9.

Turk-Adawi, K. I., & Grace, S. L. (2015). Narrative review comparing the benefits of and participation in cardiac rehabilitation in high-, middle and low-income countries. *Heart Lung and Circulation*, *24*(5), 510–520. http://doi.org/10.1016/j.hlc.2014.11.013

Turk-Adawi, K., Supervia, M., Lopez-Jimenez, F., Pesah, E., Ding, R., Britto, R. R., … Grace, S. L. (2019). Cardiac rehabilitation availability and density around the globe. *EClinicalMedicine*, *13*, 31–45. http://doi.org/10.1016/j.eclinm.2019.06.007

Vale, M. J., Jelinek, M. V., Grigg, L. E., & Newman, R. W. (2003). Coaching patients on achieving cardiovascular health (COACH): A multicenter randomized trial in patients with coronary heart disease. *Arch Intern Med.*, *163*, 2775–2783. http://doi.org/10.1001/archinte.163.22.2775

Vosbergen, S., Janzen, J., Jan, P., Zwieten, M. C. B. Van, Lacroix, J., Idema, K., … Peek, N. (2013). A qualitative participatory study to identify experiences of coronary heart disease patients to support the development of online self-management services. *International Journal of Medical Informatics*, *82*(12), 1183–1194. http://doi.org/10.1016/j.ijmedinf.2013.09.001

Wang, W., Jiang, Y., He, H.-G., & Koh, K. W. L. (2016). A randomized controlled trial on the effectiveness of a home-based self-management programme for community-dwelling patients with myocardial infarction. *European Journal of Cardiovascular Nursing*, *15*(6), 398–408. http://doi.org/http://dx.doi.org/10.1177/1474515115586904

Wannamethee, S. G., Shaper, A. G., Lennon, L., & Morris, R. W. (2005). Metabolic syndrome vs Framingham risk score for prediction of coronary heart disease, stroke, and type 2 diabetes mellitus. *Arch Intern Med.*, *165*, 2644–2650. http://doi.org/10.1001/archinte.165.22.2644

Wiedemann, A. U., Lippke, S., Reuter, T., Schu, B., Ziegelmann, J. P., & Schwarzer, R. (2009). Prediction of stage transitions in fruit and vegetable intake. *Health Education*

Research, *24*(4), 596–607. http://doi.org/10.1093/her/cyn061
Wilson, P. W. F., D'Agostino, R. B., Levy, D., Belanger, A. M., Silbershatz, H., & Kannel, W. B. (1998). Prediction of coronary heart disease using risk factor categories. *Circulation*, *97*, 1837–1847. http://doi.org/10.1161/01.CIR.97.18.1837
World Health Organization. (2017). Cardiovascular diseases: Key facts. Retrieved from https://www.who.int/news-room/fact-sheets/detail/cardiovascular-diseases-(CVD)
World Health Organization. (2018). Deaths by cause, age, sex, by country and by region, 2000-2016. Retrieved from http://www.who.int/healthinfo/global_burden_disease/estimates/en/.
World Health Organization. (2018). Physical activity: Key facts. Retrieved from https://www.who.int/news-room/fact-sheets/detail/physical-activity
World Medical Association. (2013). World Medical Association Declaration of Helsinki: Ethical principles for medical research involving human subjects. *JAMA*, *310*(20), 2191–2194. http://doi.org/10.1001/jama.2013.281053
Zarski et al. (2018). Turning good intentions into actions by using the health action process approach to predict adherence to internet-based depression prevention: Secondary analysis of a randomized controlled trial. *J Med Internet Res*, *20*(1), e9. http://doi.org/10.2196/jmir.8814
Zengin, E., Bickel, C., Schnabel, R. B., Zeller, T., Lackner, K., Rupprecht, H.-J., … Westermann, D. (2015). Risk factors of coronary artery disease in secondary prevention-Results from the atherogene study. *PLoS ONE*, *10*(7), 1–13. http://doi.org/10.1371/journal.pone.0131434
Zhang, P., Hu, Y., Xing, F., Li, C., Lan, W., & Zhang, X. (2017). Effects of a nurse-led transitional care program on clinical outcomes, health-related knowledge, physical and mental health status among Chinese patients with coronary artery disease: A randomized controlled trial. *International Journal of Nursing Studies*, *74*, 34–43. http://doi.org/10.1016/j.ijnurstu.2017.04.004
Zheng, X., Spatz, E. S., Bai, X., Huo, X., Ding, Q., Horak, P., … Krumholz, H. M. (2019). Effect of text messaging on risk factor management in patients with coronary heart disease: The CHAT randomized clinical trial. *Circulation: Cardiovascular Quality & Outcomes*, *12*, e005616–e005616. http://doi.org/10.1161/CIRCOUTCOMES.119.005616

Appendix A. Subject Information Sheet

The effectiveness of a motivated, action-based intervention on improving physical activity level, exercise self-efficacy and cardiovascular risk factors of coronary heart disease patients: A randomized controlled trial

Dear participants,

I am Mr. Karthikesu Karthijekan, currently studying PhD at Faculty of Medicine, The Chinese University of Hong Kong, Hong Kong SAR. You are sincerely invited to take part in a research study titled **"The effectiveness of a motivated, action-based intervention on improving physical activity level, exercise self-efficacy and cardiovascular risk factors of coronary heart disease patients: A randomized controlled trial".**

This study is aimed to enhance the physical activity level, and exercise self-efficacy of CHD patients to improve the health outcomes such as body weight, body mass index, waist circumference, waist-hip ratio, lipid levels, blood glucose, and blood pressure through a hospital plus home-based motivated, action-based intervention that included exercise (brisk walking) and education using the developed exercise protocol and education manual. The findings of this study could provide evidence on an effective and safe strategy to improve the cardiovascular risk factors of CHD patients.

This is an experimental study in which you will be randomly assigned either to the intervention group or control group. If you are assigned to the intervention group, you will receive motivated, action-based intervention that included exercise and education using the exercise protocol and education manual for 12 weeks after discharge from the hospital in addition to the usual care is provided by the hospital. You are asked not to show and share the contents of the exercise protocol and education manual with anyone who has had CHD since others might have some different problem that needs different management and the contents of the manuals might not be suitable for them. On the other hand, if you are assigned to the control group, you will receive the usual care provided by the hospital. All participants will be invited to complete the questionnaires and health check-up at baseline, at immediate post-intervention (12 weeks after study entry), and one-month post-intervention (16 weeks after study entry).

Your participation in this study is voluntary. If you decide not to participate or withdraw from the study, you may do so at any time without loss of medical care or any other available treatment for your illness or condition. Any information obtained in connection with this study will remain strictly confidential and at no time will the participant's name to appear in any report of this study. You are free to ask any questions or to clarify any queries regarding this study at any time from the researcher (Mr. Karthikesu Karthijekan, PhD Student, Contact No: 0772029816, email: jeshikarthi@gmail.com).

Thank you for your support and participation in this study.

Yours faithfully,
Mr. Karthikesu Karthijekan, PhD Student,
Faculty of Medicine,
The Chinese University of Hong Kong,
Hong Kong. SAR.

Appendix B. Informed Consent form

The effectiveness of a motivated, action-based intervention on improving physical activity level, exercise self-efficacy and cardiovascular risk factors of coronary heart disease patients: A randomized controlled trial

In signing this consent, I am giving my consent to participate in the research titled **"The effectiveness of a motivated, action-based intervention on improving physical activity level, exercise self-efficacy and cardiovascular risk factors of coronary heart disease patients: A randomized controlled trial"** is conducted by Mr. Karthikesu Karthijekan, PhD student of Faculty of Medicine, The Chinese University of Hong Kong, Hong Kong SAR.

I understand that this study will be based on the experimental method that I will be randomly assigned either to the test group or the control. If I am selected for the test group, I will receive the hospital plus home-based motivated, action-based intervention that included exercise (brisk walking) and education using the exercise and education manual for 12 weeks after discharge from the hospital in addition to usual care is provided by the hospital. I have been informed not to disseminate the information about the intervention, not to show and discuss the education manual and exercise protocol with "other CHD patients" since others might have a different problem that the information in the intervention manual might not be suitable for them. I will be asked to follow the exercise and education manual for 12 weeks and to ask for the response to the questionnaires at baseline, 3 months, and 4 months after study entry. On the other hand, if I am assigned to the control group, I will receive the usual care provided by the hospital. Further, I will be also asked to complete the questionnaires independently.

I have been informed that all information that I provided will be kept confidential and will not be given to anyone else. I was guaranteed that all information I provided will be used only for research purposes and will be destroyed after five years from the study completion. I am aware that my participation is voluntary and I am free to withdraw from this study anytime without penalty of any kind.

I confirmed that I have read and have been informed of the study purpose, the procedure that will undergo and, the risk and benefit that I may experience. Alternatives to my participation have also been discussed. All my questions and personal concerns on all the above issues have been asked and fully explained by the researcher. I have read and understood this consent form. Therefore, I agree to give my consent to participate in this study.

Name of the participant: ………………………………………………………………………..

Signature of the participant: …………………………................ Date: ....

Name of research personnel: ………………………………………………………………...

Signature of research personnel: ……………………………..... Date: ………………………

Appendix C. Flow diagram of the study protocol

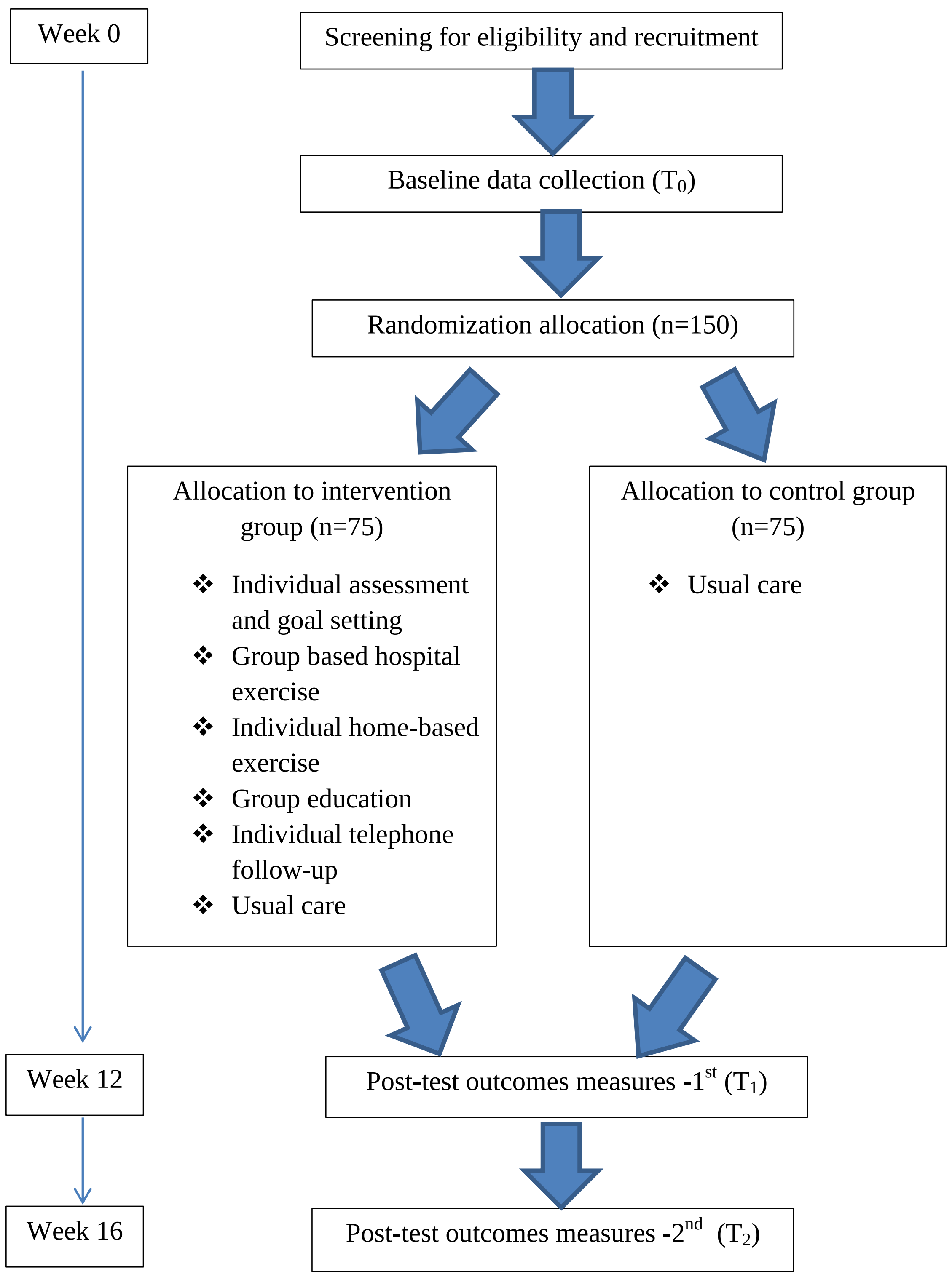

Fig. 1 Flow diagram of the study protocol

Appendix D. Study questionnaire **Date:**

Confidential **Serial No** ☐☐☐

The data is collected only for the study purpose. Answer all questions. (Please indicate (X) mark in the appropriate box and indicate your answer in the given space.)

Socio-demographic Characteristics

01. Sex: a) Male ☐ b) Female ☐

02. What is your age? _______ **(In years)**

03. What is your marital status?
    a) Single ☐ b) Married ☐
    c) Widowed ☐ d) Divorced/Separated ☐

04. What is your Ethnicity?
    a) Tamil ☐ b) Sinhalese ☐
    c) Moor ☐ d) Burger ☐

05. What is your education level?
    a) Never attended school ☐ b) Primary education ☐
    c) Secondary education ☐ d) Diploma ☐
    e) Degree ☐ f) Postgraduate ☐

06. What is your occupation?
    a) Unemployed ☐ b) Self-employee ☐ c) Government ☐
    d) Nongovernment employee ☐ e) Other ___________________________

07. What is your monthly income in Rupees?
    a) Less than 15,000 ☐ b) 15,000-29,999 ☐
    c) 30,000-50,000 ☐ d) More than 50,000 ☐

Risk factors for coronary heart disease (CHD)

08. Have you ever smoked?
    a) Yes ☐ **(go to Q.09)** b) No ☐ **(go to Q.11)**

09. Are you currently smoking?
    a) Yes ☐ **(go to Q.10)** b) No ☐ **(go to Q.11)**

10. How long you have to be smoking?
    a) Less than 1 year ☐ b) 1-3 years ☐
    c) 4-6 years ☐ d) 7-10 years ☐
    e) More than 10 years ☐

11. Have you ever drunk?

a) Yes ☐ **(go to Q.12)** b) No ☐ **(go to Q.14)**

12. Are you currently drinking?

a) Yes ☐ **(go to Q.13)** b) No ☐ **(go to Q.14)**

13. How long you have been drinking?

a) Less than 1 year ☐ b) 1-3 years ☐
c) 4-6 years ☐ d) 7-10 years ☐
e) More than 10 years ☐

14. Are you?

a) Vegetarian ☐ b) Non vegetarian ☐

1. Are you regularly using drugs?

a) Yes ☐ b) No ☐

16. What are the drugs you use?

…………………………………..
…………………………………..
…………………………………..
…………………………………..

Anthropometry and Investigation Measures

17.Weight **(Kg)**:-_________ 18. Height **(cm)**:-____________ 19. BMI **(Kg/m²)**: ________

20. Waist circumference **(cm)**: ________ 21. Hip circumference **(cm)**: ____________

22. Waist Hip Ratio:-_________

23. Blood pressure **(mmHg)** at baseline:

I. Systolic bllod pressure (SBP) ___________
II. Diastolic blood pressure (DBP) ___________

24. Fasting blood glucose level **(mg/dl)** at baseline: **(check medical record)**

I. ___________

25. **Lipid profile at baseline: (check medical record)**

a) Triglyceride level **(mg/dl)** ……………………………….

b) High-Density Lipoprotein (HDL) **(mg/dl)** ………………………

c) Low-Density Lipoprotein (LDL) **(mg/dl)** ………………………..

d) Total Cholesterol level **(mg/dl)** …………………………………………

e) HDL: LDL ………………………………………………...

Thank you for your cooperation

Appendix E

The translated Tamil version of Cardiac Exercise Self-efficacy Instrument (CESEI)

இதய உடற்பயிற்சி சுயசெயல்திறன் அளவீட்டு கருவி

தயவுசெய்து கீழே குறிப்பிடப்பட்டுள்ள செயற்பாடுகளை செய்வதற்கு நீங்கள் எந்தளவுக்கு தன்னம்பிக்கை கொண்டுள்ளீர்கள் என்பதை பிரதிபலிக்கும் இலக்கத்தை சுற்றி வட்டமிடுக.

	மிகக்குறைவான தன்னம்பிக்கை		சாதாரண தன்னம்பிக்கை		மிகஅதிகமான தன்னம்பிக்கை
1. உடற்பயிற்சி செய்ய ஆரம்பிக்கும் முன் உடல் ரீதியாக தயாராகுதல்.	1	2	3	4	5
2. நெஞ்சுவலி ஏற்படாதவாறு உடற்பயிற்சி செய்தல்.	1	2	3	4	5
3. உடற்பயிற்சி செய்யும் அளவு அதிகரித்து விட்டதை உணர்ந்து அதனை நிறுத்துவது தொடர்பாக அறிந்திருத்தல்.	1	2	3	4	5
4. அசௌகரியமான நிலையிலும் உடற்பயிற்சி செய்யும் இயலுமை.	1	2	3	4	5
5. உடற்பயிற்சியின் முன்னரும் உடற்பயிற்சியின் பின்னரும் எனது இதய துடிப்பு வீதம் என்னவாக இருக்க வேண்டும் என அறிந்து வைத்திருத்தல்.	1	2	3	4	5
6. உடற்பயிற்சியின் பின்னர் உடலை சாதாரண நிலைக்கு தளர்த்துதல்.	1	2	3	4	5
7. அதிக வேலைப்பளு உள்ள நாட்களிலும் உடற்பயிற்சியை ஒரு அங்கமாக வைத்திருத்தல்.	1	2	3	4	5
8. தாங்கிக்கொள்ளக்கூடிய கடுமையான உடற்பயிற்சி செய்தல்.	1	2	3	4	5
9. எந்த உடற்பயிற்சி எனக்கு ஆரோக்கியமானது என்பதை அறிந்திருத்தல்.	1	2	3	4	5
10. எந்த சந்தர்ப்பத்தில் எனது உடற்பயிற்சியின் அளவினை அதிகரிக்கலாம் என அறிந்திருத்தல்.	1	2	3	4	5
11. தாங்கிக்கொள்ளக்கூடிய சற்று கடினமான உடற்பயிற்சி செய்தல்.	1	2	3	4	5
12. உடற்பயிற்சியின் முன்னரும் உடற்பயிற்சியின் பின்னரும் எனது இதய துடிப்பு வீதத்தை அளத்தல்.	1	2	3	4	5
13. வைத்தியசாலையில் அனுமதிக்கப்படுவதற்கு முன்னரான காலத்தில் இருந்த செயற்பாட்டின் அளவிற்கு மீள திரும்புதல்.	1	2	3	4	5
14. தாங்கிக்கொள்ளக்கூடிய இலகுவான உடற்பயிற்சி செய்தல்.	1	2	3	4	5
15. குறைந்தது வாரத்திற்கு மூன்று தடவைகள் இருபது நிமிடங்களிற்கு குறையாமல் உடற்பயிற்சி செய்தல்.	1	2	3	4	5
16. வீட்டில் சுயமாகவே உடற்பயிற்சி செய்தல்.	1	2	3	4	5

Appendix F

The translated Tamil version of International Physical Activity Questionnaire Short Form (IPAQ-SF)

அனைத்துலக உடல் செயல்பாடு கேள்வித்தாள் – குறும்படிவம்

கடந்த ஏழு நாட்களின் போது நீங்கள் ஈடுபட்ட அனைத்து **தீவிர** உடற்செயற்பாடுகள் பற்றி சிந்திக்கவும். **தீவிர** உடற்செயற்பாடுகள் எனப்படுவது யாதெனில் கடினமான உடற்தொழிற்பாட்டை கொண்டவையும், வழமையைவிட கடினமாக சுவாசிக்க வைப்பவையுமான உடற்தொழிற்பாடுகளாகும். அவ்வாறாக ஒரே தடவையில் குறைந்தது பத்து நிமிடங்களாவது ஈடுபட்ட உடற்செயற்பாடுகளை பற்றி மட்டுமே சிந்திக்கவும்.

1. **கடந்த ஏழு நாட்களில்**, எத்தனை நாட்களில் நீங்கள் **தீவிர** உடற்செயற்பாடுகளில் அதாவது கனமான பொருட்களை தூக்குவது, குழி தோண்டுதல், சீரான மூச்சடக்காமல் செய்யும் தீவிரமான உடற்பயிற்சிகள் அல்லது வேகமாக சைக்கிள் ஓட்டுவது போன்றவற்றில் ஈடுபட்டீர்கள்?

   வாரமொன்றிற்கு .... **நாட்கள்**

   ☐ தீவிர உடற்செயற்பாடுகளில் ஈடுபடவில்லை எனில் **வினா இலக்கம் 3ற்கு செல்க.**

2. அவ்வாறான நாட்களில் நாளொன்றிற்கு வழமையாக எவ்வளவு நேரம் இந்த **தீவிர** உடற்செயற்பாடுகளில் செலவிட்டீர்கள்?

   நாளொன்றிற்கு .... **மணித்தியாலங்கள்**

   நாளொன்றிற்கு .... **நிமிடங்கள்**

   ☐ தெரியாது/நிச்சயமில்லை

கடந்த ஏழு நாட்களின் போது நீங்கள் ஈடுபட்ட அனைத்து **மிதமான** உடற்செயற்பாடுகள் பற்றி சிந்திக்கவும். **மிதமான** உடற்செயற்பாடுகள் எனப்படுவது யாதெனில் மிதமான உடற்தொழிற்பாட்டை கொண்டவையும், வழமையைவிட சற்று கடினமாக சுவாசிக்க வைப்பவையுமான உடற்தொழிற்பாடுகளாகும். அவ்வாறாக ஒரே தடவையில் குறைந்தது பத்து நிமிடங்களாவது ஈடுபட்ட உடற்செயற்பாடுகளை பற்றி மட்டுமே சிந்திக்கவும்.

3. **கடந்த ஏழு நாட்களில்**, எத்தனை நாட்களில் நீங்கள் **மிதமான** உடற்செயற்பாடுகளில் அதாவது இலேசான பொருட்களை தூக்குவது, சாதாரணமாக சைக்கிள் ஓட்டுவது அல்லது இரட்டை டெனிஸ் விளையாடுவது போன்றவற்றில் ஈடுப்பட்டீர்கள்? நடப்பதை உள்ளடக்க வேண்டாம்.

   வாரமொன்றிற்கு .... **நாட்கள்**

   ☐ மிதமான உடற்செயற்பாடுகளில் ஈடுபடவில்லை எனில் **வினா இலக்கம் 5ற்கு செல்க.**

4. அவ்வாறான நாட்களில் நாளொன்றிற்கு வழமையாக எவ்வளவு நேரம் இந்த **மிதமான** உடற்செயற்பாடுகளில் செலவிட்டீர்கள்?

   நாளொன்றிற்கு .... **மணித்தியாலங்கள்**

   நாளொன்றிற்கு .... **நிமிடங்கள்**

   ☐ தெரியாது/நிச்சயமில்லை

கடந்த ஏழு நாட்களில் நீங்கள் **நடப்பதற்கு** செலவிட்ட நேரத்தை பற்றி சிந்திக்கவும். இது வேலையின் போது, வீட்டில், ஒரு இடத்திலிருந்து இன்னும் ஒரு இடத்திற்கான பிரயாணத்தின் போது, மற்றும் பொழுதுபோக்கிற்காக, விளையாட்டு போட்டிகளில், உடற்பயிற்சிற்காக, அல்லது ஓய்வு நேர நடவடிக்கைகளுக்காக நடந்தவற்றை உள்ளடக்கும்.

5. **கடந்த ஏழு நாட்களில்**, எத்தனை நாட்களில் தொடர்ச்சியாக குறைந்தது பத்து நிமிடங்களாவது **நடந்தீர்கள்**?
வாரமொன்றிற்கு .... **நாட்கள்**
☐ **நடக்கவில்லை** எனில் **வினா இலக்கம் 7ற்கு செல்க.**

6. அவ்வாறான நாட்களில் நாளொன்றிற்கு **நடப்பதற்கு** எவ்வளவு நேரம் செலவிட்டீர்கள்?
நாளொன்றிற்கு .... **மணித்தியாலங்கள்**
நாளொன்றிற்கு .... **நிமிடங்கள்**
☐ தெரியாது/நிச்சயமில்லை

இறுதிவினாவானது **கடந்த ஏழு நாட்களில்** வாரநாட்களில் நீங்கள் அமர்ந்திருந்த நேரத்தை பற்றியதாகும். இது தொழிலில், வீட்டில், கல்வி நடவடிக்கையில் மற்றும் ஒய்வு நேரம் போன்றவற்றில் அமர்ந்திருந்ததை உள்ளடக்கும். மேலும் இது மேசையின் முன் அமர்ந்து வேலை செய்யும் நேரம், நண்பர்களை பார்ப்பது, வாசித்தல், அல்லது அமர்ந்து மற்றும் படுத்து தொலைக்காட்சி பார்க்கும் நேரத்தையும் உள்ளடக்கலாம்.

7. **கடந்த ஏழு நாட்களில்**, வாரநாள் ஒன்றில் எவ்வளவு நேரம் அமர்ந்திருந்தீர்கள்?
நாளொன்றிற்கு .... **மணித்தியாலங்கள்**
நாளொன்றிற்கு .... **நிமிடங்கள்**
☐ தெரியாது/நிச்சயமில்லை
